# Resolving the boron anomaly in SrF_2_-borate glasses using combined MRN-TCT-percolation framework

**DOI:** 10.1038/s41598-025-10662-2

**Published:** 2025-10-28

**Authors:** Enas Abd El-Raouf, Ahmed Hamalawy, Sameh Hassan

**Affiliations:** Physics Department, Faculty of Science, Menoufia University, Shebin El-Koom, Menoufia 32511 Egypt

**Keywords:** Fluorine-modified borate glass, Tetrahedral borate units (B^4+^) and N4 ratio, Non-bridging oxygen bonds (NBOs), Modified random network (MRN), Topological constraint theory (TCT), Percolation theory, UV–vis and ultrasonic velocities, Materials science, Nanoscience and technology, Optics and photonics, Physics

## Abstract

This study resolves the longstanding “boron anomaly” by integrating Modified Random Network (MRN), Topological Constraint Theory (TCT), and Percolation Theory. SrF_2_ (acting dually as network disruptor/charge compensator) was substituted in 50B_2_O_3_–(20–X)PbO–(X)SrF_2_–20CaO–10ZnO glasses (X = 0–20 mol%) via melt-quenching technique. MRN reveals SrF_2_-induced phase separation into ${\text{F}}^{-}$-rich disordered domains (XRD: 45° hump) and B^4+^-rich ordered domains (28° hump), explaining increased Urbach energy (from 0.291 to 0.389 eV). TCT quantifies (B^3+^ to B^4+^) conversion (N4% increase from 33.8% to 77.9%), where percolating BO_4_ tetrahedra enhance rigidity. This increases Young’s modulus (from 61.98 to 83.25 GPa) despite density loss and widens direct (from 2.964 to 3.215 eV) and indirect (from 2.537 to 2.652 eV) bandgaps. Percolation Theory identifies a critical threshold at 10 mol% SrF_2_: below this, ${\text{F}}^{-}$ disrupts the network (forming BO_2_F_2_/BO_3_F defects), causing non-monotonic N4% (minimum 27.3% at X = 5 mol%) and modulus anomalies. Above 10 mol%, ${\text{F}}^{-}$ saturation enables charge compensation (stabilizing BO_4_⁻ units). This triple-theory synergy decodes the anomaly and enables eco-friendly glass design with tailored opto-mechanical properties.

## Introduction

For nearly a century, the Continuous Random Network (CRN) model has been the primary method for representing the structure of glass. It depicts a completely random yet uniformly connected network, as described by Zachariasen^1,2^.

The ‘boron anomaly’, a century-old concept in borate glasses, manifests as contradictory property evolution: network modifiers reduce density yet enhance rigidity^3^. This defies the Continuous Random Network (CRN) model^1,2^, which treats disorder as homogeneous and fails to explain medium-range heterogeneities (e.g., phase-separated domains) or percolation-driven thresholds. Consequently, predicting and designing functional glasses remains challenging.

Current frameworks address fragments of glass behavior: MRN describes medium-range disorder models, modifier clustering, but neglects rigidity constraints^4^. TCT predicts rigidity transitions via bond constraints (B^3+^:3 constraints/atom → flexible; B^4+^:4 constraints/atom → rigid) but assumes homogeneity^5,6^. While percolation theory models connectivity thresholds, but ignores atomic-scale topology^7^. However, no existing framework synergistically resolves SrF_2_-induced anomalies. A unified approach is critical to understanding how the composition affects properties.

Heavy metal oxides like PbO optimize properties (enhance boron’s tetrahedral coordination, improving rigidity and optical properties)^8–10^. However, PbO introduces toxicity and environmental hazards, optical and electronic drawbacks, and structural and thermal limitations^11^, demanding eco-friendly alternatives. Strontium fluoride (SrF_2_) emerges as a promising candidate, impacting glass network connectivity through a dual role^12,13^. Firstly by replacing bridging oxygen (BOs) with non-bridging fluoride as BO_2_F_2_ and BO_3_F units via defect generator^14,15^. Also, they are integrated into the borate network and induce structural changes, converting some B^3+^ to B^4+^ units via network polymerization. These structural modifications influence the physical and structural properties of borate glasses^16,17^.

In this study, we resolve these problems by integrating MRN-TCT- percolation theories for the first time into a unified framework. The main purpose of the article is to discuss how SrF_2_’s dual role drives disorder-to-order transitions. Why percolation thresholds trigger property anomalies. And how to harness this synergy for eco-friendly glasses with tailored opto-mechanical properties through the glass’s physical, FTIR, Ultrasonic velocities and UV–Vis characteristics.

## Experimental details

### System preparation

The samples of mixed strontium fluoride with leadborate glasses were prepared with the composition 50B_2_O_3_ + (20-X) PbO + (X)SrF_2_ + 20CaO + 10ZnO in mole fractions, with X varying from 0 to 20 mol%. The chemicals used for glass synthesis were high-purity Boron Oxide (B_2_O_3_) (99% PIOCHEM), Lead Oxide (PbO) (95% LOBA CHEMIE PVT.LTD), Strontium Fluoride (SrF_**2**_) (97% RIEDEL–DE HAEN AG), Calcium Oxide (CaO) (> 98% Fluka AG), and Zinc Oxide (ZnO) (99.5% AppliChem). The composition batch was prepared using a conventional melting-quenching technique. The mixture was ground and then melted for 25 min in a crucible at 1000 °C for X = 0 mol%, with a 25 °C increase for each subsequent X mol%, as shown in Table 1. After 20 min, the melts were shaken and rotated to ensure homogeneity and remove bubbles. After an additional 5 min, the melts were poured onto a marble plate and then transferred to an annealing furnace set at 350 °C for 2 h and allowed to cool down to room temperature in the furnace.

**Table 1:** Melting temperature and the composition of created glasses. (PW is the final prepared disk of the present glass system).

B_2_O_3_	PbO	SrF_2_	ZnO	CaO	Temperature	Sample	P.W
50	20	0	10	20	1000 °C	X = 0	
50	15	5	10	20	1025 °C	X = 5	
50	12.5	7.5	10	20	1050 °C	X = 7.5	
50	10	10	10	20	1075 °C	X = 10	
50	5	15	10	20	1100 °C	X = 15	
50	0	20	10	20	1125 °C	X = 20	

The samples that initially tested X = 0, 5, 10, 15 and 20 mol%. When density and molar volume between X = 5 mol% and X = 10 mol% dropped and sharply increased, respectively, an additional sample was forced to be prepared at X = 7.5 mol% to clarify if this was a gradual shift or abrupt threshold.

### Characterization techniques

#### Density and molar volume

Using the conventional Archimedes method, the density of the glass samples was calculated. Toluene was used as an inert immersion liquid, and a sensitive decimal four digital balance was used for these experiments. The density was calculated using the following relation^3^:

$$\uprho=\frac{{\text{W}}_{\text{a}}}{{\text{W}}_{\text{a}}-{\text{W}}_{\text{b}}}\times {\uprho }_{\text{t}}$$

where W_a_ is the glass weight in the air, W_b_ is the glass weight in toluene, and ${\uprho }_{\text{t}}$ The density of toluene and equal to 0.863 g/cm^3^.

The molar volume (V_m_) of all the prepared glasses is the volume of one mole of that prepared sample and is calculated as given by the relation^3^:

$${\text{V}}_{\text{m}}=\frac{\text{MW}}{\uprho }$$

where MW is the mean molecular weight and ρ is the experimental density of prepared glasses.

#### X‑ray diffraction (XRD)

The samples investigated were submitted to X-ray diffraction (XRD) examinations at room temperature using a Brucker 2D Phase 2nd gen-X-ray diffractometer equipped with a graphite monochromatic for Cu-K-alpha-radiation (λ = 1.54178 Å) with source power of 30 kV and 10 mA.

#### Infrared spectroscopy (FTIR)

Using a Bruker ALPHA II FTIR Spectrophotometer, the glasses’ FTIR absorption spectra in the 1600–400 cm^−1^ spectral region were acquired. These spectra were used to examine structural alterations induced by SrF_2_ addition to the glass network. And also, identify the functional groups of boron oxide in glass samples, and the infrared spectra were acquired.

#### UV–Vis spectroscopy

One practical method for determining the direct and indirect band gaps is UV–Vis spectroscopy. A single-beam UV–Vis spectrophotometer (Acculab USA) was used to measure the diffuse absorption spectra of bulk glass samples at room temperature in the wavelength range of 190–1100 nm.

#### Ultrasonic velocities measurements

The ultrasonic velocities were measured at room temperature using the pulse-echo technique, where the time intervals between two echoes were recorded with the Krautkramer USM 36 L model. This was done by measuring the elapsed time between the start and the receipt of the pulse as displayed on the screen.

## Results and discussion

### MRN-driven disorder

#### X-ray diffraction

**Fig. 1:**
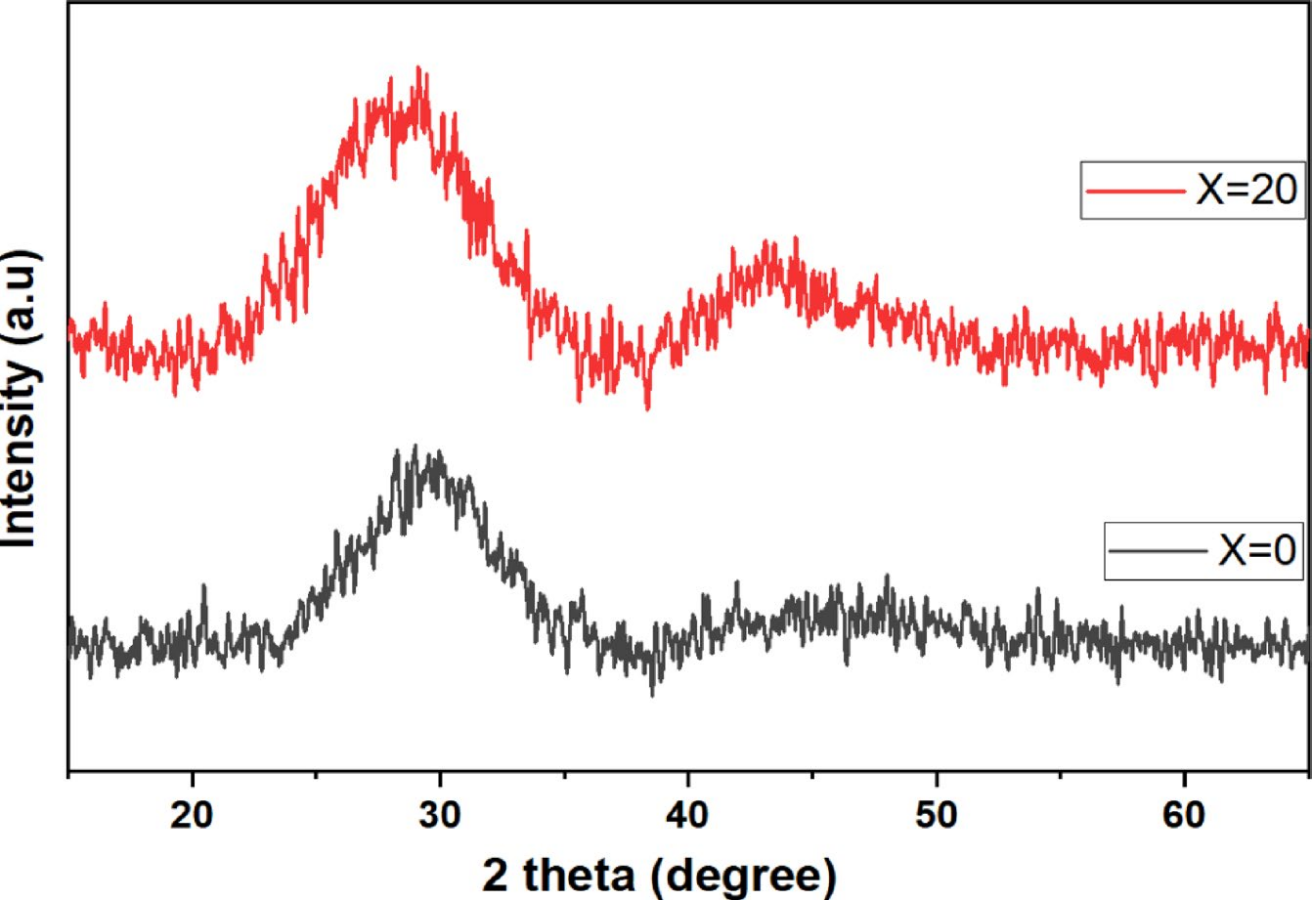
XRD spectra as the function of the glass concentration of SrF_2_ for (X = 0 and 20 mol %) for 50B_2_O_3_–(20X) PbO–(X)SrF_2_–20CaO–10ZnO (where X = 0, 5, 7.5, 10, 15, and 20 mol%).

The XRD patterns of SrF_2_-modified glasses confirm the amorphous nature of the glasses. And also show two large humps at low (2θ = 28°) and high (2θ = 45°) scattering angles shown in Fig. 1, demonstrating phase-separated medium-range order within the amorphous matrix. The low-angle hump associated with polymerized BO_4_ tetrahedral is caused by coherent scattering across rigid B-O-B networks^14,18,19^. The high-angle hump indicates disordered regions with ${\text{F}}^{-}$ ions and Sr^2+^-${\text{F}}^{-}$ clusters, consistent with the Modified Random Network (MRN) theory.

#### Physical parameters

The physical characteristics of the produced glass samples, shown in Table 2, provide quantitative evidence for MRN-predicted atomic-scale disorder evolution through three interconnected mechanisms^12,19^.

**Table 2:** Physical characteristics of the produced glass samples, as well as the SrF_2_ concentration, were measured.

(Physical Parameters)/X	Sample	X = 0	X = 5	X = 7.5	X = 10	X = 15	X = 20
Density (g/cm^3^)	$\text{P}$	4.65	4.28	3.76	3.75	3.51	3.17
Molar Volume (cm^3^/mol)	${\text{V}}_{\text{m}}$	21.24	21.96	24.32	23.76	23.96	25.04
Ion Concentration of dopant “ × 10^+22^ (ions/cm^3^)”	N	0	0.41	0.56	0.76	1.13	1.44
Polaron Radius (Å)	${\text{r}}_{\text{P}}$	–	2.51	2.27	2.05	1.80	1.66
Internuclear Distance (Å)	${\text{r}}_{\text{i}}$	–	6.24	5.64	5.09	4.45	4.11
Average Boron to Boron Distance (Å)	$<{\text{d}}_{\text{B}-\text{B}}>$	3.272	3.31	3.42	3.39	3.41	3.47
Volume of Boron network per mole	${\text{V}}_{\text{m}}^{\text{B}}$	21.24	21.96	24.32	23.77	23.96	25.04

- Density Collapse

Table 2 shows that as the SrF_2_ concentration rises, the density falls from 4.65 g/cm^3^ to 3.17 g/cm^3^. This decreases results from network depolymerization due to non-bridging oxygen (NBO) formation in the basic glass network that generates interstitial voids through two MRN-validated pathways. And also, smaller ${\text{F}}^{-}$ creates fluorinated sites when substituting bridging oxygen^17,18^. These voids are characteristic of MRN’s modifier-rich domains

$$B-O-B+{Sr}^{2+}\to B-O-{Sr}^{2+}-F+B-{O}^{-} (NBOs)$$

(b) Volumetric Expansion

Table 2 illustrates that the molar volume increases from 21.24 cm^3^/mol to 25.04 cm^3^/mol as the concentration of SrF_2_ increases^18^. This increase is caused by a decrease in the coulomb interaction of a greater concentration of fluoride ions^20^. As well as the addition of fluoride ions creates voids and forms a more open structure (NBOs).

**Fig. 2:**
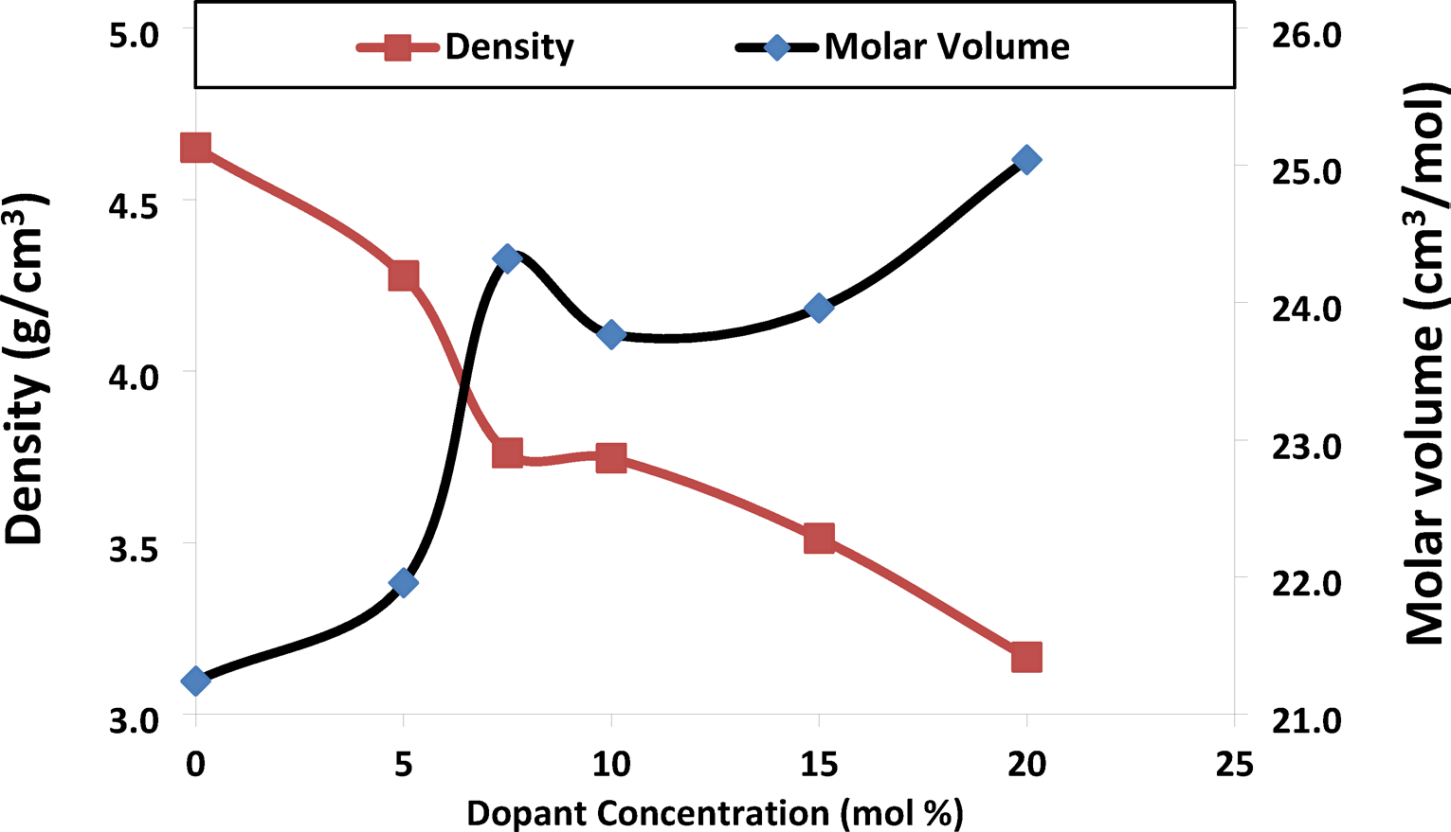
molar volume V_m_ and density ρ as a function of the glass concentration of SrF_2_ for 50B_2_O_3_–(20–X) PbO–(X)SrF_2_–20CaO–10ZnO (where X = 0, 5, 7.5, 10, 15, and 20 mol%).

The variations in density and molar volume with different SrF_2_ concentrations are shown in Fig. 2. It is clear that when SrF_2_ concentration increases, the density falls while the molar volume rises. A negative correlation and structural transformation were found between strontium fluoride concentration X = 7.5 and both density and molar volume. As a result, the replacement of oxygen ions (${\text{O}}^{2-}$) with fluoride ions (${\text{F}}^{-}$) have a smaller ionic radius (1.33 Å) compared to oxygen ions (${\text{O}}^{2-}$) (1.40 Å), creating interstitial voids. These voids create disorderly short regions that have lower density and expand the borate matrix.

Threshold behavior at the concentration of strontium fluoride X = 10. Where the structural rearrangement occurs as ${\text{F}}^{-}$ ions reach a critical concentration (saturation) where they act as charge compensators for B^4+^^16,17^.

(c) Average Boron–Boron Distance

The boron-boron distance expansion reveals medium-range disorder, calculated by the following equation^19^:

$$<{\text{d}}_{\text{B}-\text{B}}>={\left[\frac{{\text{V}}_{\text{m}}^{\text{B}}}{{\text{N}}_{\text{A}}}\right]}^\frac{1}{3}$$

where N_A_ is Avogadro’s number and ${\text{V}}_{\text{m}}^{\text{B}}$ represents the volume of boron atoms per mole and is given by the following equation^19^:

$${\text{V}}_{\text{m}}^{\text{B}}=\frac{{\text{V}}_{\text{m}}}{2\left(1-{\text{X}}_{\text{B}}\right)}$$

where ${\text{V}}_{\text{m}}$ is the molar volume and X_B_ is the mole fraction of borate glass.

**Fig. 3:**
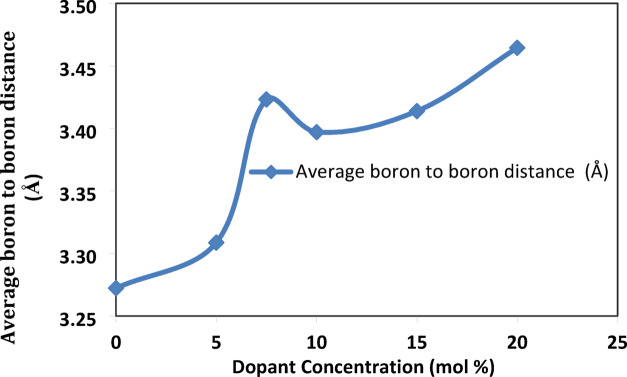
the variation in the average boron to boron distance of the prepared glass system with the SrF_2_ concentration for 50B_2_O_3_–(20–X) PbO–(X)SrF_2_–20CaO–10ZnO (where X = 0, 5, 7.5, 10, 15, and 20 mol%).

**Fig. 4:**
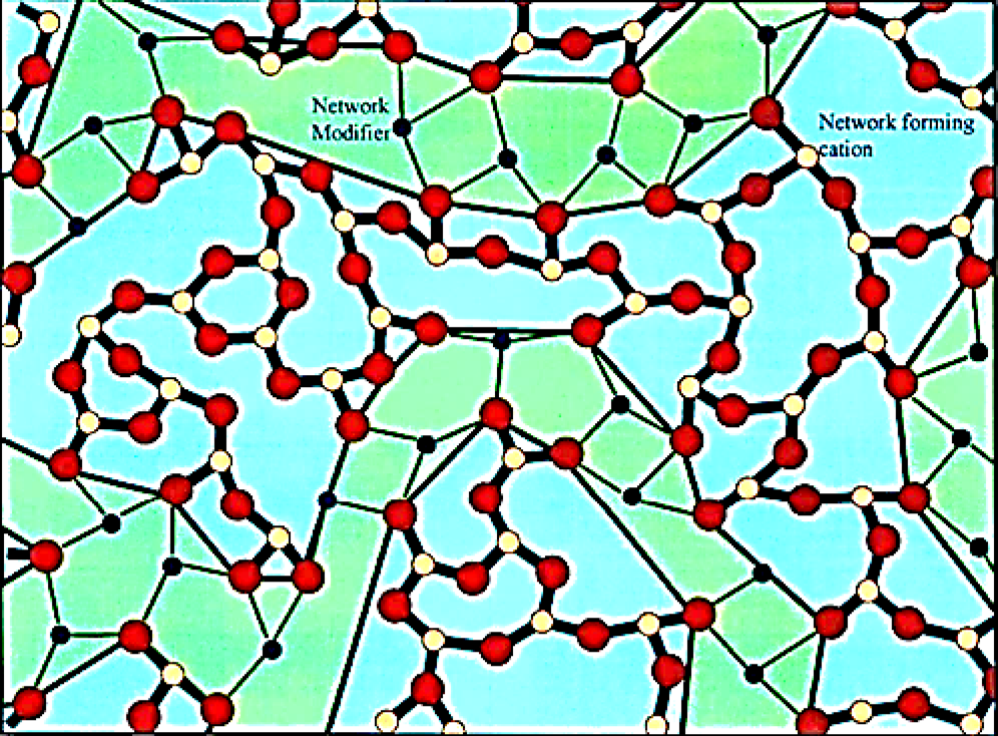
the phase separation, percolation, and cluster formation of the glass system^20^.

Based on the previously discussed calculations, Fig. 3 shows that as the concentration of SrF_2_ increases, the average boron-boron distance (< d_B−B_ >) also increases. This rise proves the dual role of fluoride ions as being charge compensation as well as network disruption^19^. This indicates the formation of NBOs for both (BO_**3**_F and BO_**2**_F_**2**_) units as a network disruption. At the concentration of SrF_**2**,_ X = 7.5, while the increase in molar volume, the cluster creates short-range disorder voids, expanding the network (phase separation percolation), shown in Fig. 4^7^. At concentration of SrF_**2**_ at X = 10, fluoride ions ${\text{F}}^{-}$ reach a saturation state where they act as charge compensation, enabling B^4+^ stabilization (threshold behavior).

#### OPTICAL parameters

Optical parameters, as presented in Table 3, include various glass properties such as, refractive index, dielectric and optical dielectric constants, reflection loss, molar and electronic polarizability, molar refractivity, metallization and oxygen packing density^12,19^.

**Table 3:** Measured values of the optical properties of the prepared glass system along with the concentration of SrF_2_^19^.

Optical Parameters	Sample	X = 0	X = 5	X = 7.5	X = 10	X = 15	X = 20
Refractive index	$\text{n}$	2.407	2.400	2.390	2.382	2.365	2.341
Dielectric constant	$\upvarepsilon$	5.793	5.759	5.711	5.673	5.593	5.482
Optical Dielectric Constant	$\text{p}\frac{\text{dt}}{\text{dp}}$	4.793	4.759	4.711	4.673	4.593	4.482
Reflective Loss	R_L_	0.171	0.170	0.168	0.167	0.165	0.161
Molar Refractivity (cm^3^/mol)	R_m_	13.064	13.468	14.857	14.474	14.492	15.001
Molar Polarizability (Å^3^ )	${\upalpha}_{\text{m}}$	5.182	5.342	5.893	5.741	5.748	5.950
Electronic Polarizability (Å^3^ )	${\upalpha}_{\text{e}}$	0.244	0.243	0.242	0.242	0.239	0.238
Metallization	$\text{M}$	0.385	0.387	0.389	0.391	0.396	0.401
Oxygen Packing Density (g/atm)	OPD	94.157	88.807	79.161	79.950	77.216	71.885

- The refractive index (n)

The refractive index (n) is a measurement of a crucial optical characteristic of glass. The following relation is used to derive the refractive index from the optical band gap energies^19^:

$$\frac{{\text{n}}^{2}-1}{{\text{n}}^{2}+1}=1-\sqrt{\frac{\text{Eg}}{20}}$$

where Eg is the direct energy band gap.

The glass system’s dielectric constant (ε) can be expressed as^19^:

$$\upvarepsilon ={\text{n}}^{2}$$

where n is the refractive index of the prepared glass sample.

Optical dielectric constant^19^:

$$\text{p}\frac{\text{dt}}{\text{dp}}=\left(\upvarepsilon -1\right)={\text{n}}^{2}-1$$

**Fig. 5:**
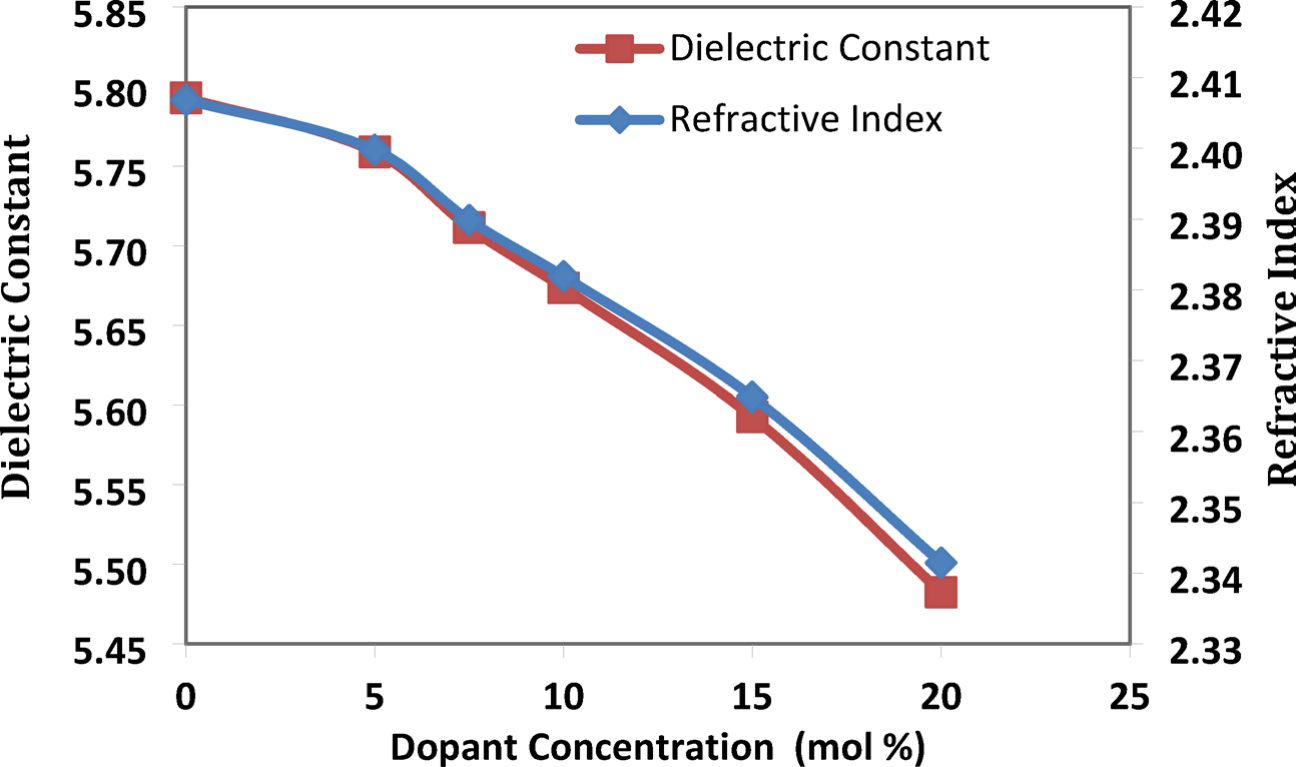
the refractive index and dielectric constant of the prepared system with the SrF_2_ concentration for 50B_2_O_3_–(20–X) PbO–(X)SrF_2_–20CaO–10ZnO (where X = 0, 5, 7.5, 10, 15, and 20 mol%).

The above mentioned equations were used to determine the refractive index and dielectric constant of the produced glass samples. As the concentration of strontium fluoride increased, both values showed a decreasing trend. That makes sense that Sr^2+^ is replacing Pb^2+^ because Sr^2+^ has a lower polarizability than Pb^2+^. As well as these results match up with the density decrease. The decrease in density^21^, along with the increase in molar volume, indicates a decrease in the refractive index as well as a decrease in the dielectric constant, as illustrated in Fig. 5.

(b) The molar refraction (R_m_) and the molar polarizability (α_m_):

The molar fraction can be defined as the speed of light is reduced in a prepared glass compared to its speed in a vacuum per unit volume, which is evaluated from the Lorentz–Lorenz equation^21^:

$${\text{R}}_{\text{m}}=\frac{{\text{n}}^{2}-1}{{\text{n}}^{2}+2}\times {\text{V}}_{\text{m}}$$

The molar polarizability refers to the polarizability of a material per mole of a substance and is calculated from the equation^22^:

$${\upalpha}_{\text{m }}=\left(\frac{3}{4\uppi {\text{N}}_{\text{A}}}\right)\times {\text{R}}_{\text{m}}$$

where N_A_ is Avogadro’s number and R_m_ is molar refraction.

**Fig. 6:**
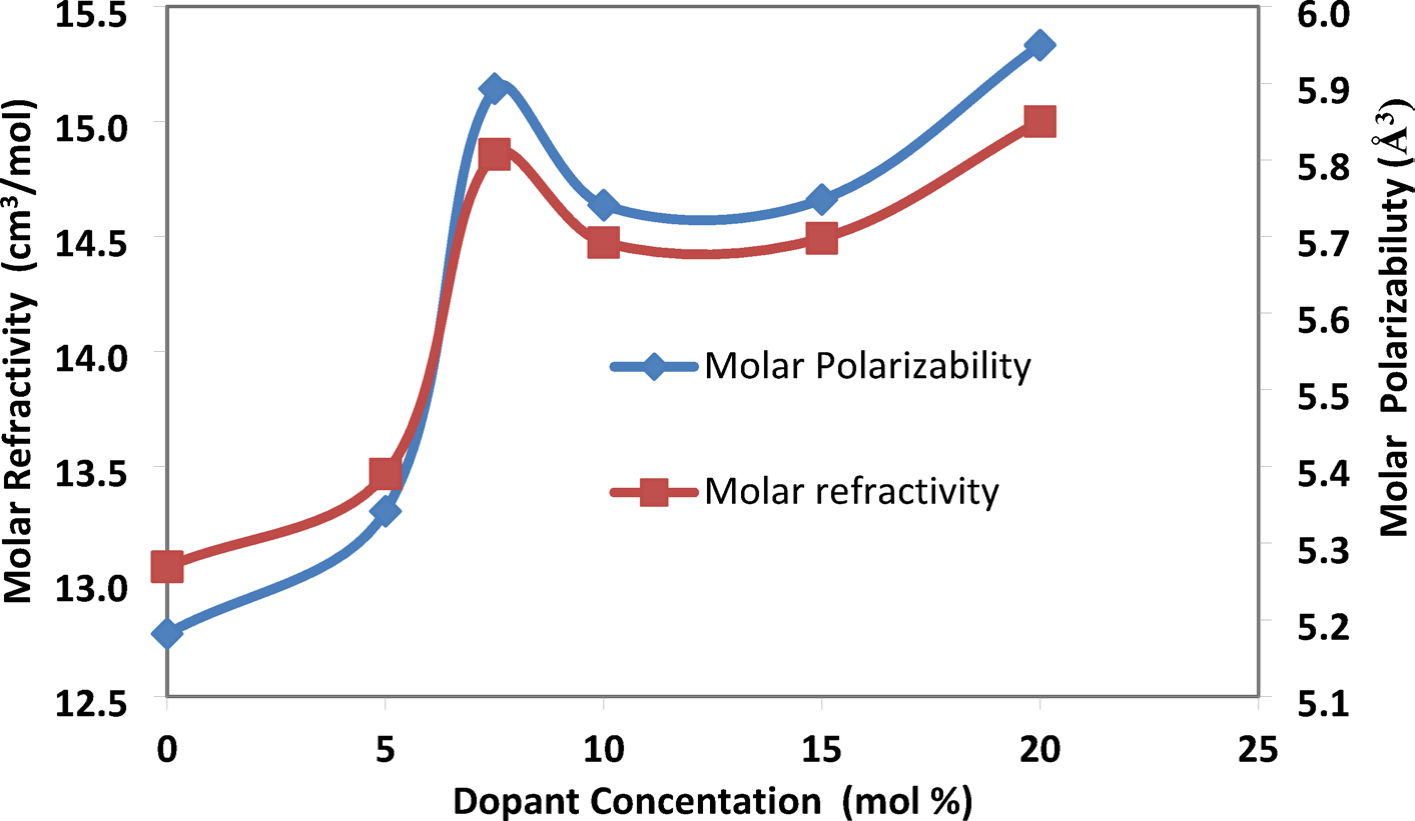
molar refractivity and molar polarizability of produced glass samples varied with the SrF_2_ concentration 50B_2_O_3_–(20–X) PbO–(X)SrF_2_–20CaO–10ZnO (where X = 0, 5, 7.5, 10, 15, and 20 mol%).

Molar polarizability as well as molar refraction increase with the rising concentration of SrF_2_ despite a decrease in refractive index. This is because the formation of tetrahedral borate units (B^4+^) as long-range-ordered charged units, which are more refractive and polarizable than trigonal borate units (B^3+^). Both molar polarizability and molar refraction depend on molar volume^17,22^. Figure 6 shows a slight deviation from the linear trend at a strontium fluoride concentration of X = 7.5, as previously discussed.

(c) Electronic polarizability

Electronic polarizability in a glass system refers to the ability of the electron cloud surrounding an atom or ion within the glass structure to distort or shift in response to an electric field. The electronic polarizability is associated with both the glass bond structure and its refractive index and has been calculated using the formula^22^:

$${\upalpha}_{\text{e}}=\frac{3\left({\text{n}}^{2}-1\right)}{4\uppi {\text{N}}_{\text{A}}\left({\text{n}}^{2}+2\right)}$$

**Fig. 7:**
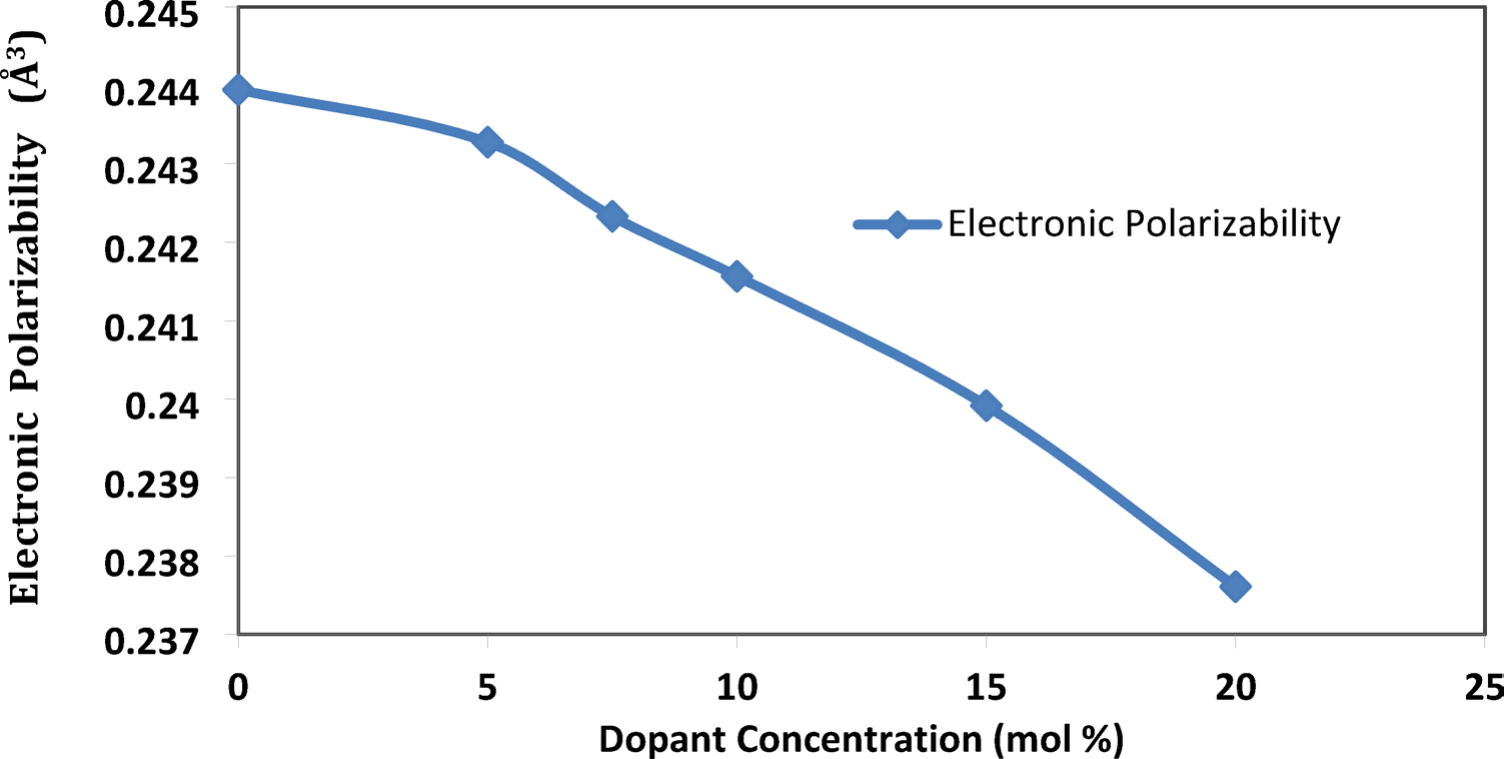
the electronic polarizability of the prepared system with the SrF_2_ concentration for 50B_2_O_3_–(20–X) PbO–(X)SrF_2_–20CaO–10ZnO (where X = 0, 5, 7.5, 10, 15, and 20 mol%).

As the concentration of fluoride ions increases, as shown in Fig. 7, the fluorinated units (BO_**3**_F, BO_**2**_F_**2**_) develop in the glass network. As well as the conversion to tetrahedral (B^4+^) units (σ-bond) lowers the electron mobility and polarizability. Thus, the electronic polarization decreases with the addition of strontium fluoride.^22^.

(d) Metallization and Reflection Loss

Glass’s ability to be either metallic or insulating, depending on the metallization criterion, is known as metallization^19^:

$$\text{M}=1-\frac{{\text{R}}_{\text{m}}}{{\text{V}}_{\text{m}}}$$

The refractive index is determined using Fresnel’s relation, which also provides information on reflection loss, which is the energy loss of incident light in the glass network and is obtained using the following formula^22^:

$${\text{R}}_{\text{L}}={\left(\frac{\text{n}-1}{\text{n}+1}\right)}^{2}$$

**Fig. 8:**
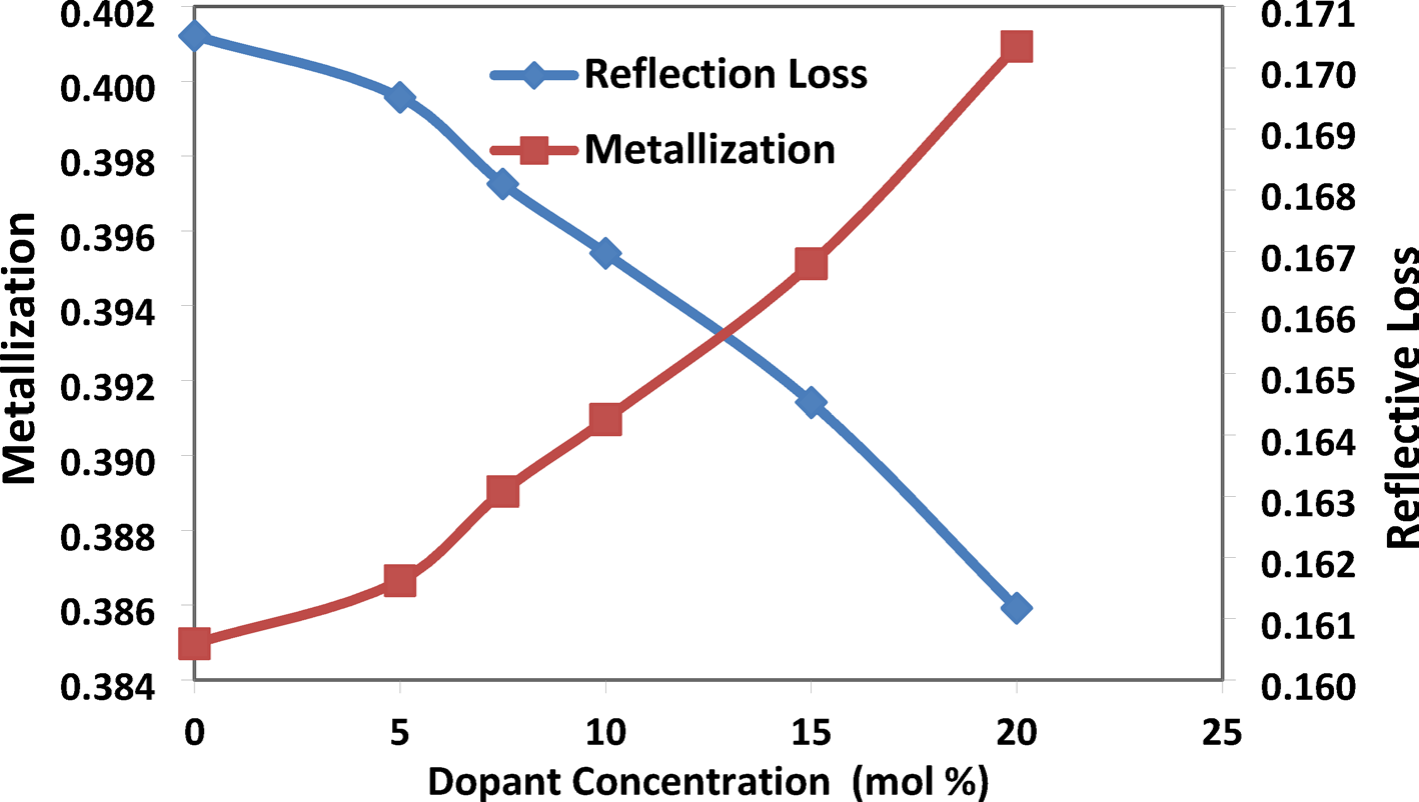
the produced glass samples with a concentration of SrF_2_ exhibit variations in metallization and reflection loss for 50B_2_O_3_–(20–X) PbO–(X)SrF_2_–20CaO–10ZnO (where X = 0, 5, 7.5, 10, 15, and 20 mol%).

Figure 8 shows that the metallization increases with the addition of strontium fluoride, for increasing the ionic behavior, and based on the theory of metallization, more ionic bonds are metallic as a result of fluorinated units (BO_**3**_F, BO_**2**_F_**2**_) bond formation^23^.

(e) The oxygen packing density (OPD)

The oxygen packing density (OPD) is another optical factor that releases light on the structure of the glass. It is determined by the relationship between the quantity of oxygen in the glass and its composition^19^:-

$$\text{OPD} ={\text{n}}_{\text{O}}\left(\frac{\uprho }{\text{MW}}\right)\times 1000$$

Where ρ is the density of the prepared glass sample and n_O_ is the number of oxygen atoms present in the glass composition.

$${n}_{O}=\sum {n}_{i} \times {O}_{i}$$

where ${n}_{i}$ is the number of moles of oxide and ${O}_{i}$ is the number of oxygen atoms per mole of oxide.

**Fig. 9:**
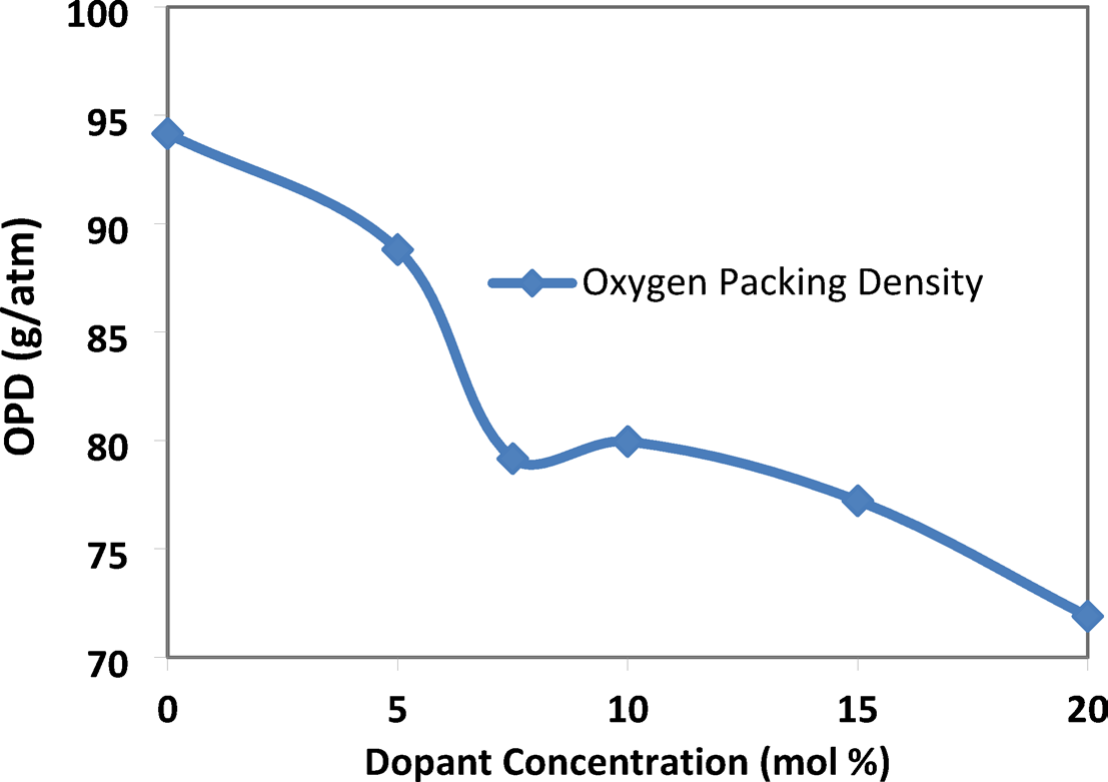
the variation in oxygen packing density of the prepared glass samples with the concentration of SrF_2_ for 50B_2_O_3_–(20–X) PbO–(X)SrF_2_–20CaO–10ZnO (where X = 0, 5, 7.5, 10, 15, and 20 mol%).

As shown in Fig. 9, due to a decrease in lead oxide percentage, some fluoride ions replace the oxygen ions present in the glass network, forming short-range-disorder units (BO_**3**_F, BO_**2**_F_**2**_)^12^.

### TCT rigidity: network constraints and rigidity percolation

#### UV-VIS: (Direct and indirect Band Gap)

**Fig. 10:**
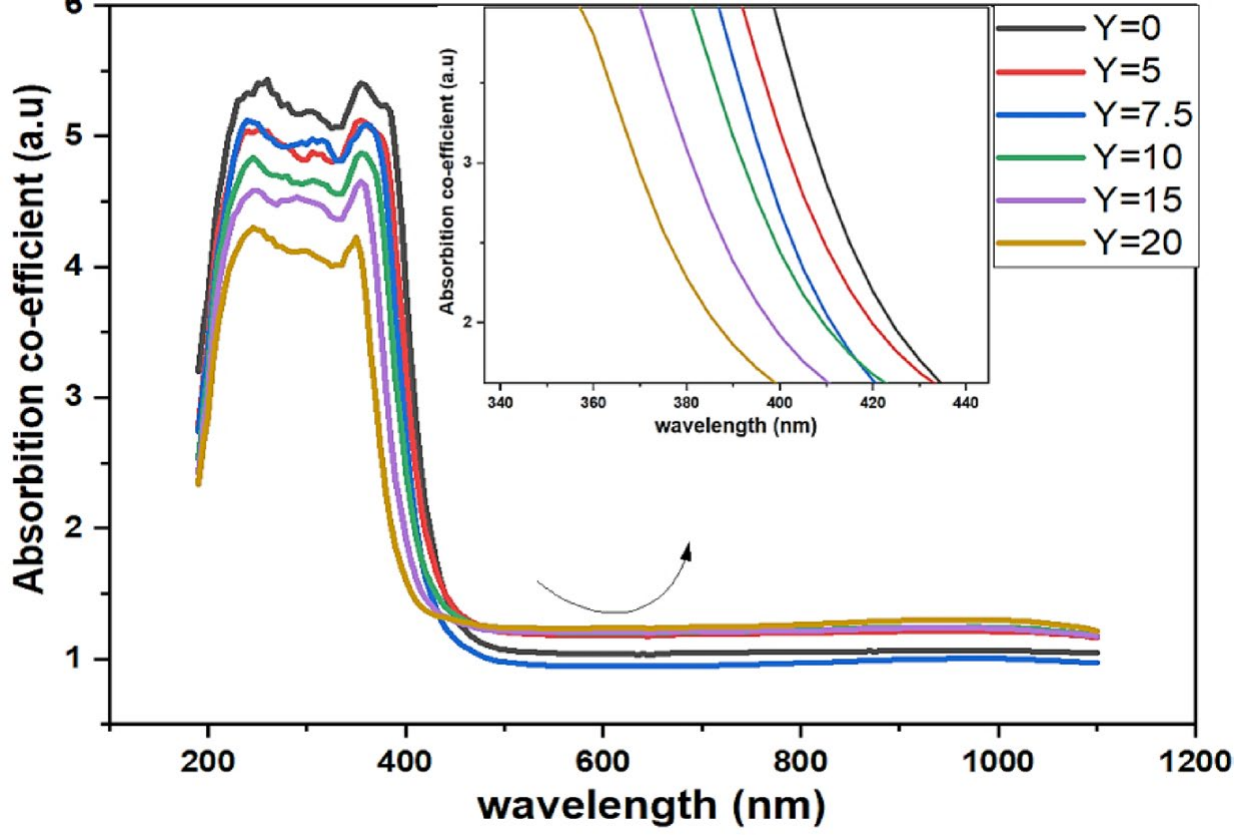
UV–Visible absorption spectra as a function of wavelength of prepared glass sample along with the SrF_2_ concentration 50B_2_O_3_–(20–X) PbO–(X)SrF_2_–20CaO–10ZnO (where X = 0, 5, 7.5, 10, 15, and 20 mol%).

The blue shift in the band edge, which continually shifts toward lower wavelengths as the SrF_2_ concentration rises, is seen in Fig. 10. Fluorine localizes electrons through strong ionic bonds, increasing the bandgap. So the creation of fluorinated units (BO_**3**_F, BO_**2**_F_**2**_) units inside the glasses is a result of the amount of fluoride ions acting as network disruption^21^.

To estimate the optical energy gaps for all samples, Davis–Mott established the following relation^2,24^:

$${\left(\alpha hv\right)}^{n}=A\left(hv-Eg\right).$$

where A is a constant, α is the absorption coefficient, Eg refers to the optical energy band gap of the sample, and hν is the photon energy of the incident spectrum. The band gap energies were figured out by using n = 2, 1/2 and plots of (αhν)^2^ and (αhν)^1/2^ for direct and indirect transitions, respectively. In low photon energy range, the width of band tails can be described by using the equation:-

$$ln\left( \alpha \right)=\text{ln}\left({\alpha }_{0}\right)+\frac{hv}{Eu}$$

where ${\alpha }_{0}$ is fixed, $\alpha$ refers to the absorption coefficient, and Eu denotes the Urbach energy*.*

**Fig. 11:**
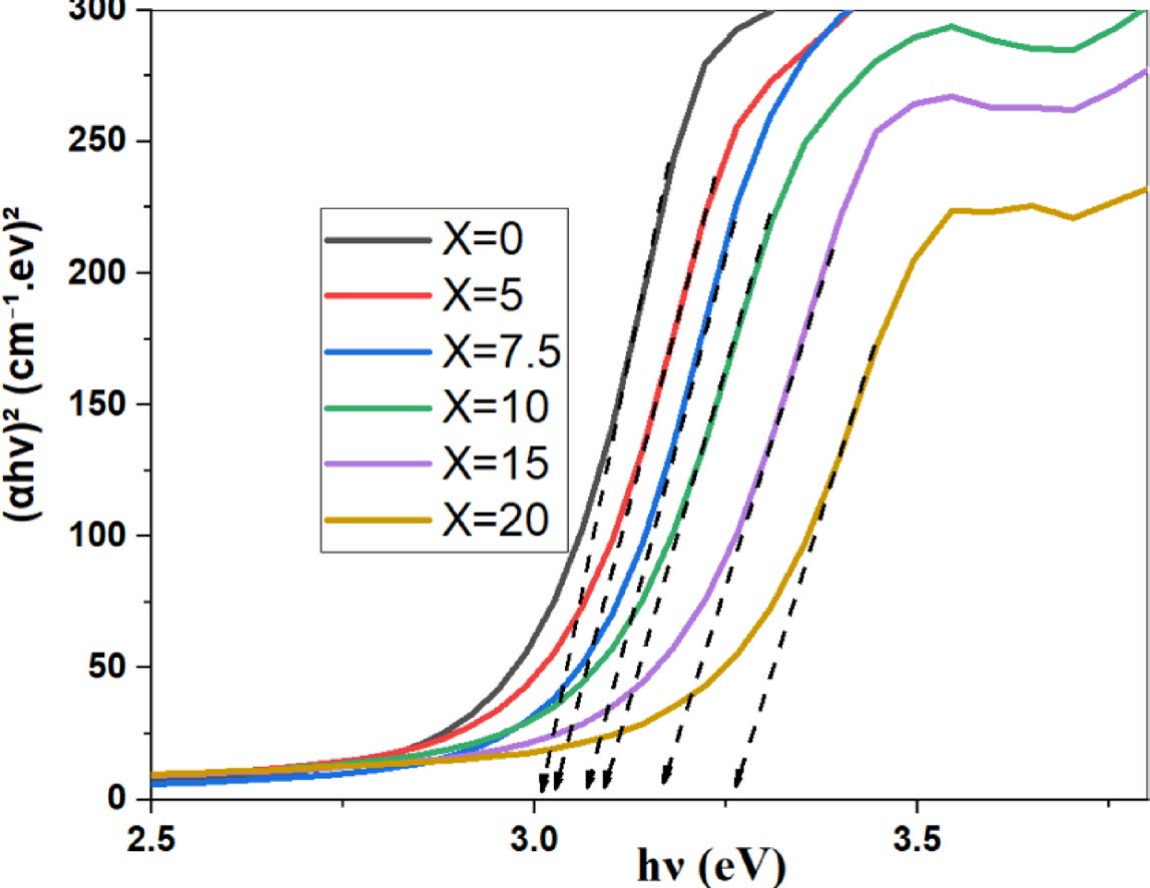
the direct energy band gap of prepared samples along with the SrF_2_ concentration for 50B_2_O_3_–(20–X) PbO–(X)SrF_2_–20CaO–10ZnO (where X = 0, 5, 7.5, 10, 15, and 20 mol%).

**Fig. 12:**
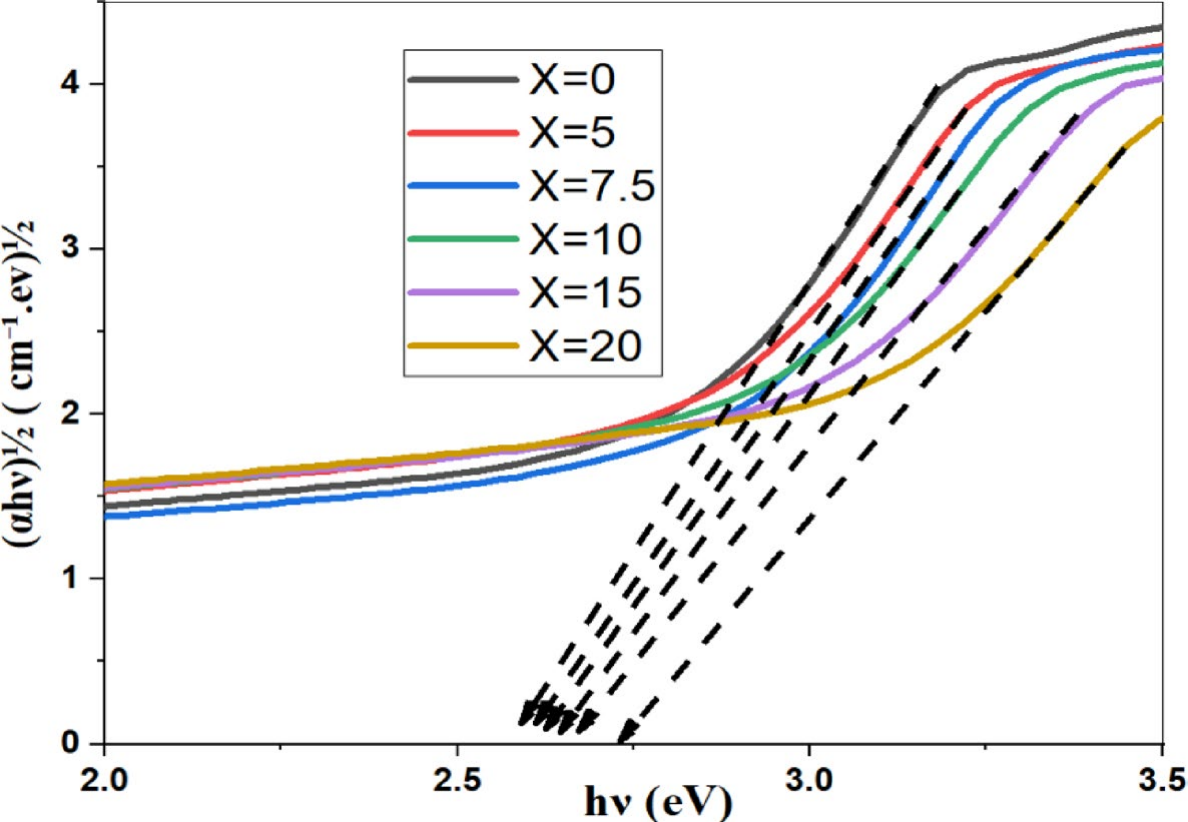
the indirect band energy gap of prepared samples along with the SrF_2_ concentration for 50B_2_O_3_–(20–X) PbO–(X)SrF_2_–20CaO–10ZnO (where X = 0, 5, 7.5, 10, 15, and 20 mol%).

**Fig. 13:**
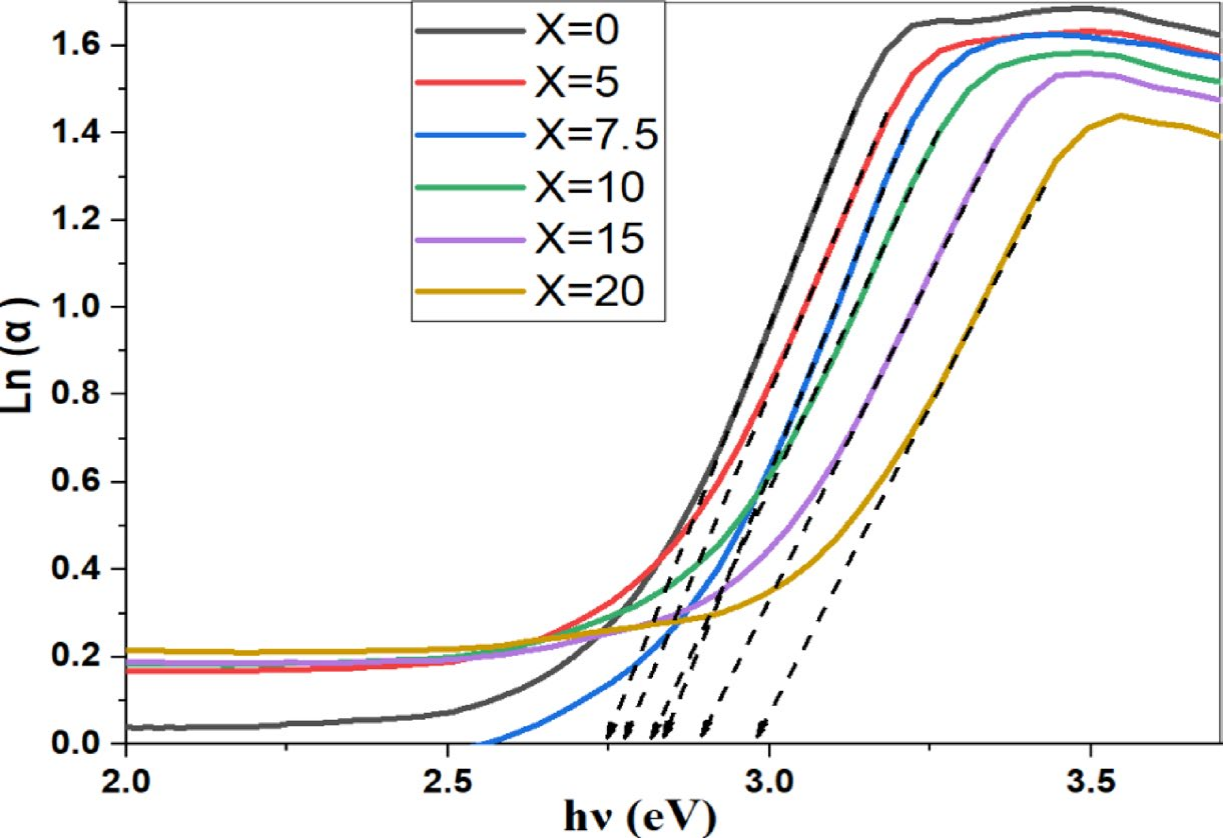
The energy urbach of prepared samples along with the SrF_2_ concentration for 50B_2_O_3_–(20–X) PbO–(X)SrF_2_–20CaO–10ZnO (where X = 0, 5, 7.5, 10, 15, and 20 mol%).

The direct, indirect band gap and Urbach energy of the prepared samples were calculated, as a function of the system composition, by the above equations and shown in Figs. 11, 12 and 13, respectively.

**Fig. 14:**
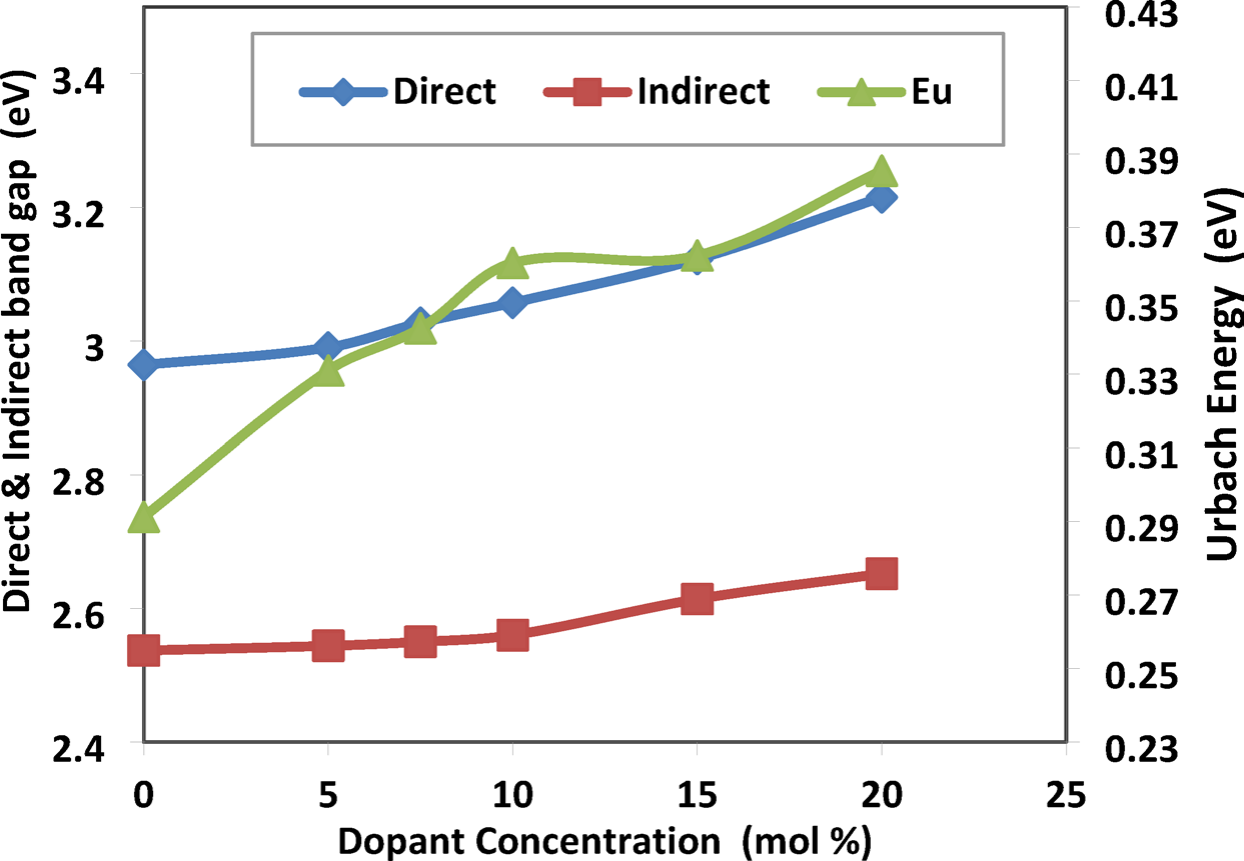
The direct, indirect band gap and Urbach energy samples with the concentration of SrF_2_ for 50B_2_O_3_–(20–X) PbO–(X)SrF_2_–20CaO–10ZnO (where X = 0, 5, 7.5, 10, 15, and 20 mol%).

TCT Rigidity studies both indirect and direct band gaps. They are found to increase with the addition of SrF_**2**_ (rising from 2.537 to 2.652 eV and from 2.964 eV to 3.215 eV, respectively) as shown in Table 4. This increase reflects BO_4_ percolation stiffening the network (for replacing Pb^2+^ with higher cation field strength Sr^2+^)^25,26^. MRN predicts the increase in Ubach energy from 0.291 eV to 0.389 eV. The model suggests an increase in short-range disorder units through the replacement of ${\text{F}}^{-}$ with ${\text{O}}^{2-}$ and ${\text{F}}^{-}$ disrupts the homogeneity and introduces voids, as shown in Fig. 14.

**Table 4:** Measured values of the energy band gap of the prepared glass system with the concentration of SrF_2_.

Dopant concentration (mol%)	Direct (eV)	Indirect (eV)	Urbach Energy (eV)
0	2.964	2.537	0.2913
5	2.99	2.544	0.331
7.5	3.027	2.55	0.3425
10	3.057	2.56	0.3603
15	3.1224	2.614	0.372
20	3.215	2.652	0.3886

#### FTIR measurements

**Fig. 15:**
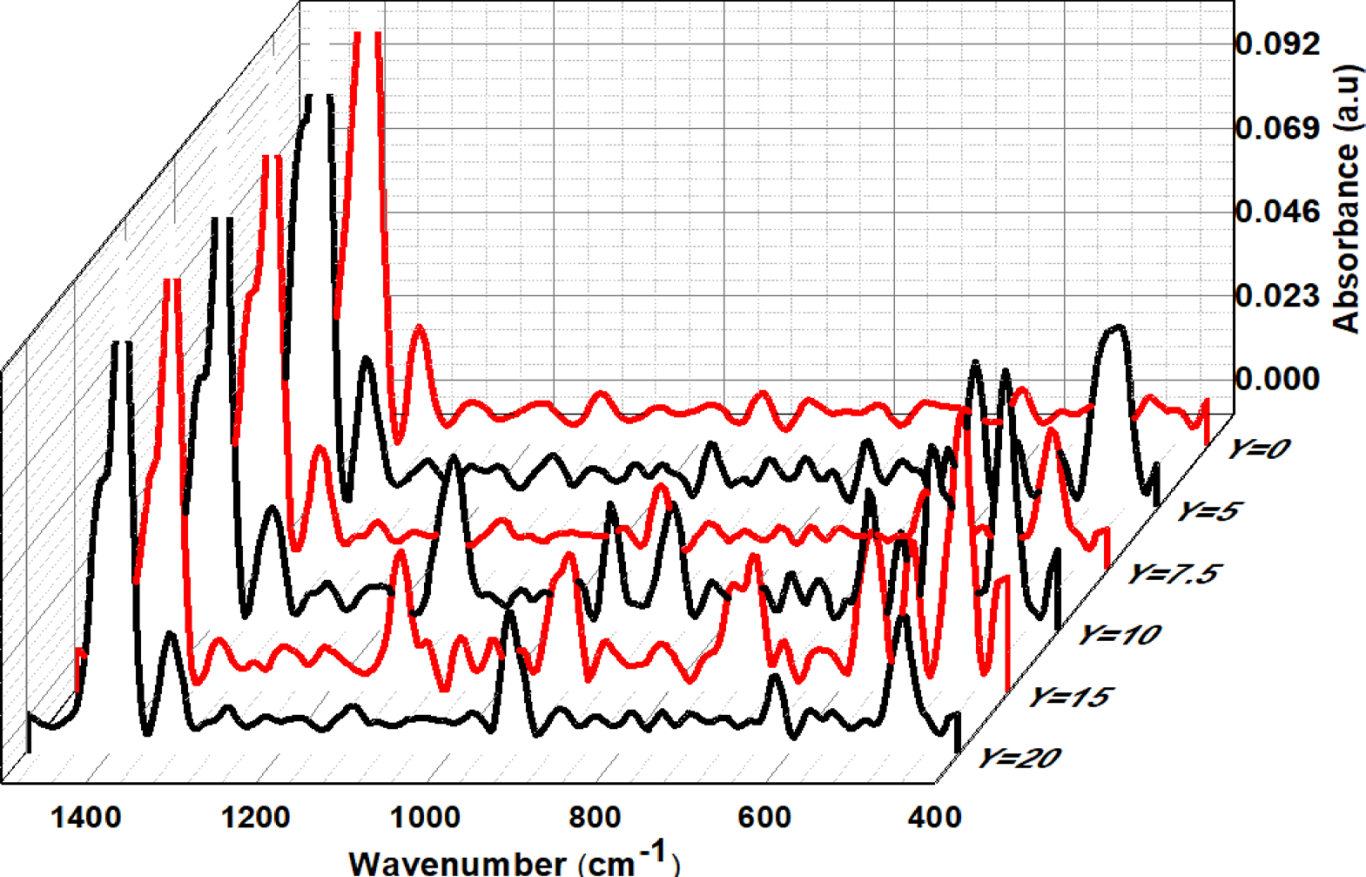
The FTIR spectra for the prepared glass system along with the concentration of SrF_2_ for 50B_2_O_3_–(20–X) PbO–(X)SrF_2_–20CaO–10ZnO (where X = 0, 5, 7.5, 10, 15, and 20 mol%).

Infrared spectroscopy is necessary to completely investigate the structure of samples in a glass system and understand how they interact with infrared light. As shown in Fig. 15, the spectra show the well-known infrared characteristic of borate glass^27^. Metal bonds are known to typically occur in the infrared spectral range of < 400–570 cm⁻^1^, with three spectral ranges associated with the borate structural unit following^28,29^. 571–767 cm^−1^ units (B^3+^), 768–1175 cm⁻^1^ (B^4+^) units, and 1176–1600 cm⁻^1^ (B^3+^) units are the first, second, and third spectral regions, respectively, discussed in Table 5.

**Table 5:** A brief assignment for each glass sample under investigation, together with information on the FTIR peak positions and the addition of strontium fluoride. (PW is present work).

References	X = 0	X = 5	X = 7.5	X = 10	X = 15	X = 20	Points to:
P.W	467	450	467	463	460	467	ZnO_4_
^5,6^	< 450						
P.W	529	519	515	526	515	510	B-O-B bending vibrations as well as borate ring angle deformations
^30^	550						
P.W	580	570	578	586	566	578	SrO bending vibrations
^12,30^	600						
P.W	628	628	628	628	628	628	Symmetric O-B-O bending vibrations of bridge oxygen in B^3+^ units
^30^	650			^12,29^	672		
P.W	690	684	682	684	698	680	stretching vibrations of pentaborate groups
^18,27^	690						
P.W	730	725	715	715	707	715	Asymmetric O-B-O bending vibrations of bridge oxygen in B^3+^ units (Boroxol Rings)
^30^	722:713						
P.W	860	860	867	860	875	868	B-O stretching vibrations of non-bridging Oxygens of tetrahedral units, (NBOs)B^4+^
^30^	833:829			^5,6^	855:879		
P.W	926	927	927	927	936	928	B-O stretching vibrations and rocking motion of tetrahedral B^4+^ units
^12^	927:928			^5,6^	960:962		
P.W	1052	1065	1077	1072	1087	1087	Stretching vibrations in pentaborate groups
^18,27^	1098:1067						
P.W	1271	1268	1263	1260	1255	1263	B-O symmetric stretching vibrations of non-bridging oxygens of trigonal units, (NBOs) B^3+^ (orthoborate groups)
^12,30^	1250:1252			^29^	1204:1226		
P.W	1331	1334	1332	1338	1338	1333	B-O asymmetric stretching vibrations of B^3+^ units, (pyroborate groups)
^30^	1365:1366						
P.W	–	1407	1410	1412	1412	1412	Stretching vibration in metaborate groups
^18^	1407						

The first region is located between 571 and 767 cm^−1^ and is associated with BO₃ units^8,29^. The symmetric and asymmetric O-B-O bending vibrations of bridging oxygen in trigonal B^3+^ units are probably responsible for the two vibrational bands in this region, which are located at 620 and 725 cm⁻^1^^18^.

The range of 768–1175 cm⁻^1^ is the intermediate spectral area^29^. Two bands are included at 860 and 927 cm⁻^1^. The rocking motion of tetrahedral B^4+^ units and B-O stretching vibrations are responsible for them. Because it was attributed to the B-O stretching vibrations of the non-bridging oxygen (NBOs) in B^4+^ units, the most significant of these bands was seen at 860 cm⁻^1^. At 927 cm⁻^1^, it was attributed to the rocking motion of tetrahedral B^4+^ units and B-O stretching vibrations^18^.

The final spectral area was assigned to B-O asymmetric/symmetric stretching vibrations of trigonal BO_3_ units after it was shown to expand around 1176–1600 cm^−1^^12,25,29^. Due to its assignment to B-O symmetric stretching vibrations of non-bridging oxygen (NBOs) in B^3+^ units^18,27^ the most significant of these bands are found at 1261 cm⁻^1^. The B-O asymmetric stretching vibrations of B^3+^ units are positioned at 1335 cm⁻^1^, shown in Table 5.

The chemical bonds in strontium fluoride are typically ionic, whereas lead oxide exhibits a higher degree of covalency. This disparity in bond character significantly impacts the strength and nature of the chemical bonds within the glass network^29^.

**Fig. 16:**
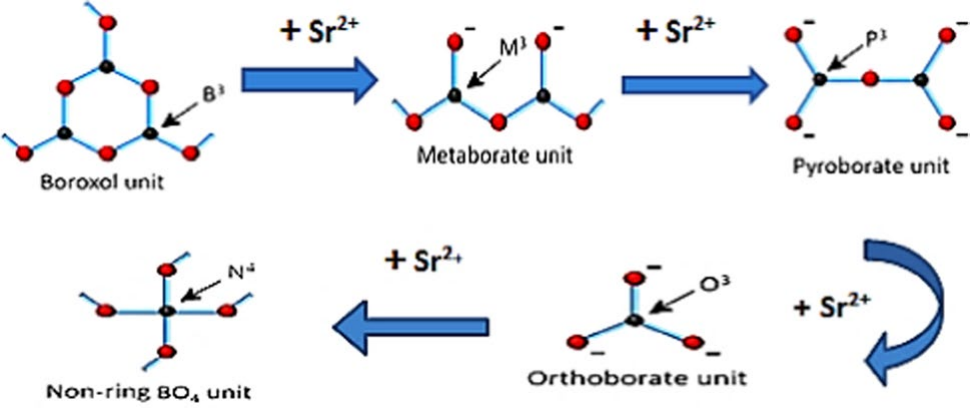
Describes the effect of Sr^2+^ on the structural groups in borate glasses.

IR spectra indicated the formation of non-bridging oxygen-containing pyroborate, orthoborate, and metaborate through weakening the boroxol rings^31,32^, due to SrF_2_ addition (Fig. 16), as shown in Table 5.

**Fig. 17:**
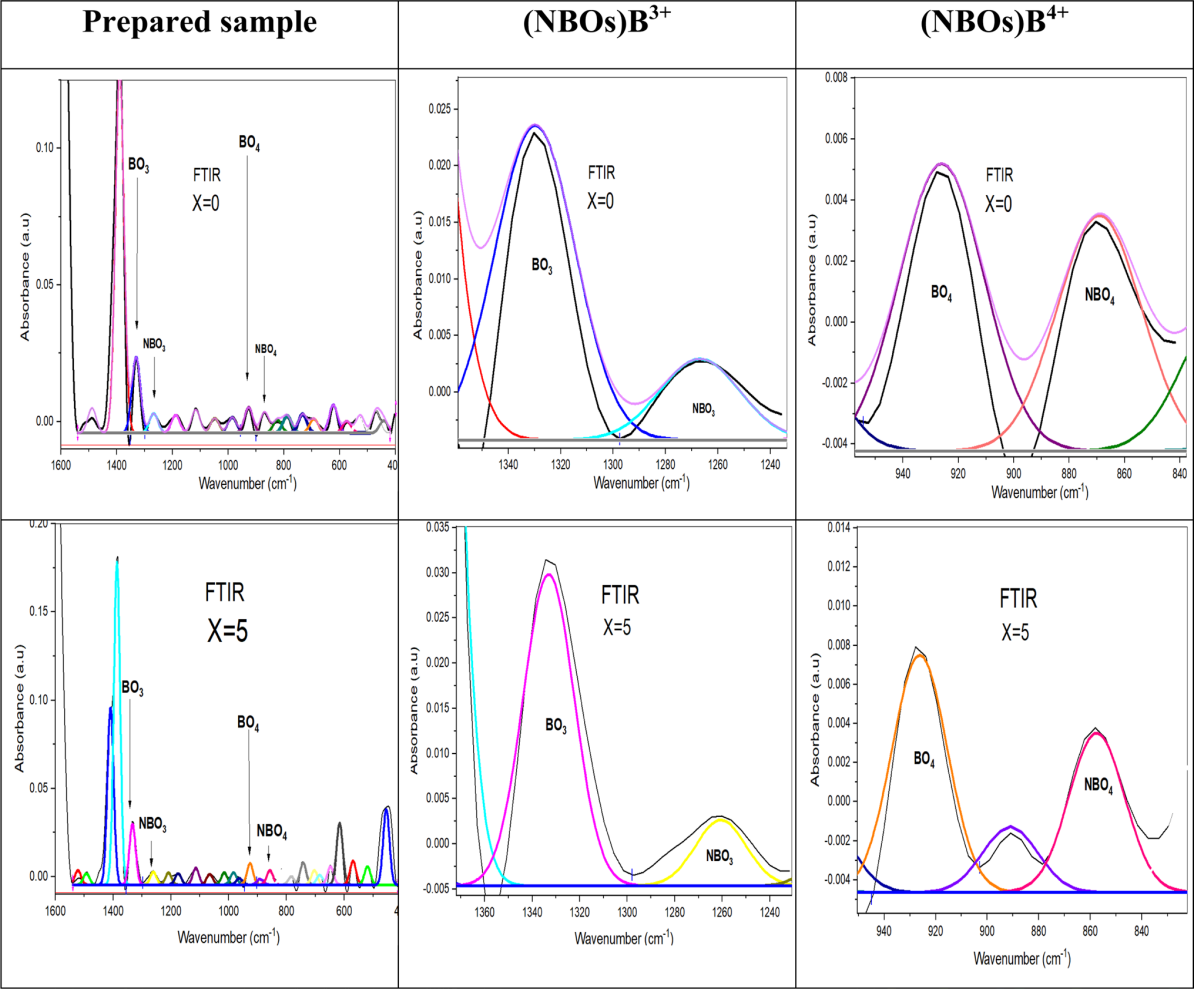
The deconvolution process of FTIR data of the prepared sample with the concentration of SrF_2_ for 50B_2_O_3_–(20–X) PbO–(X)SrF_2_–20CaO–10ZnO (where X = 0 and 5 mol%).

**Fig. 18:**
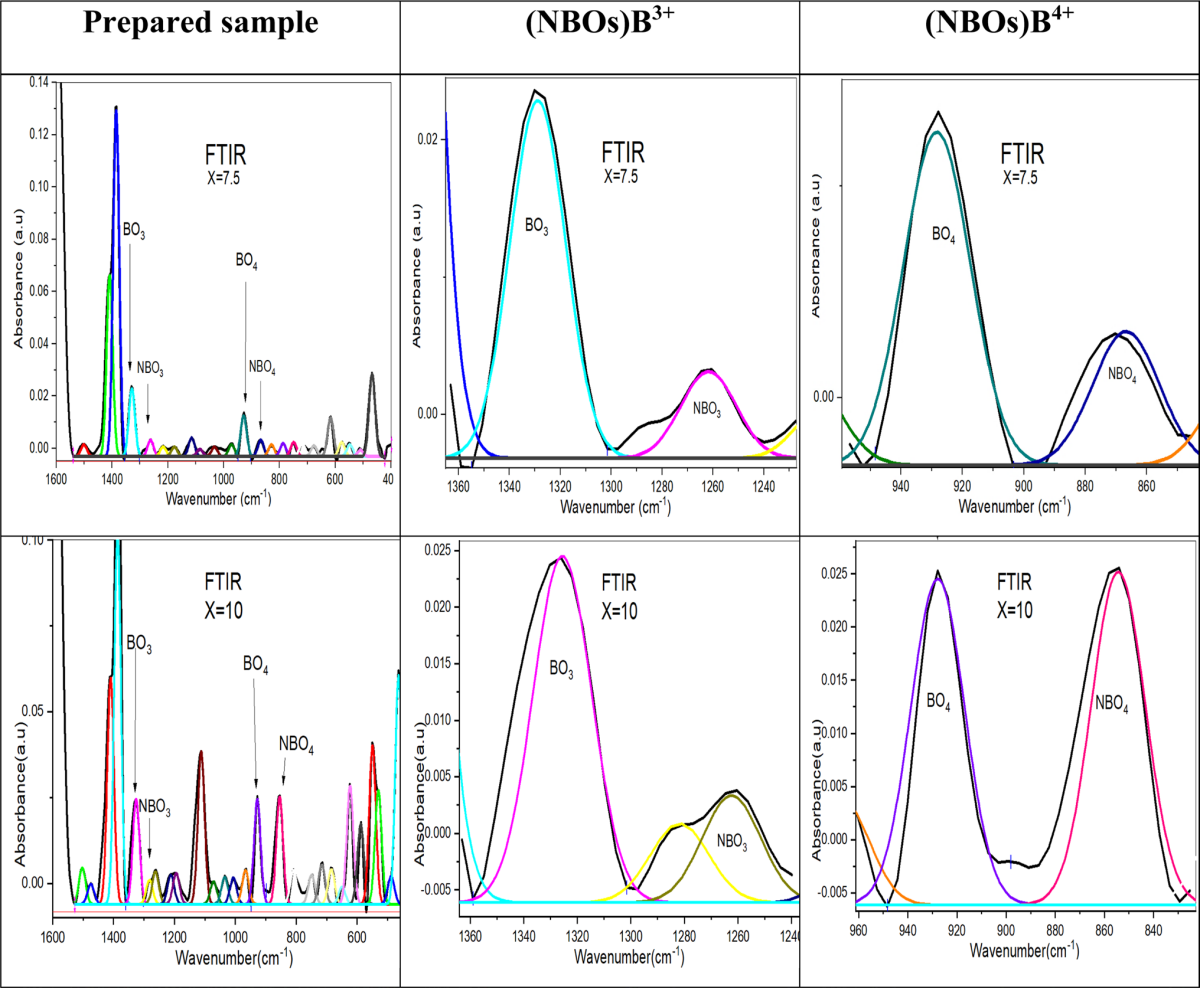
The deconvolution process of FTIR data of the prepared sample with the concentration of SrF_2_ for 50B_2_O_3_–(20–X) PbO–(X)SrF_2_–20CaO–10ZnO (where X = 0 and 5 mol%).

**Fig. 19:**
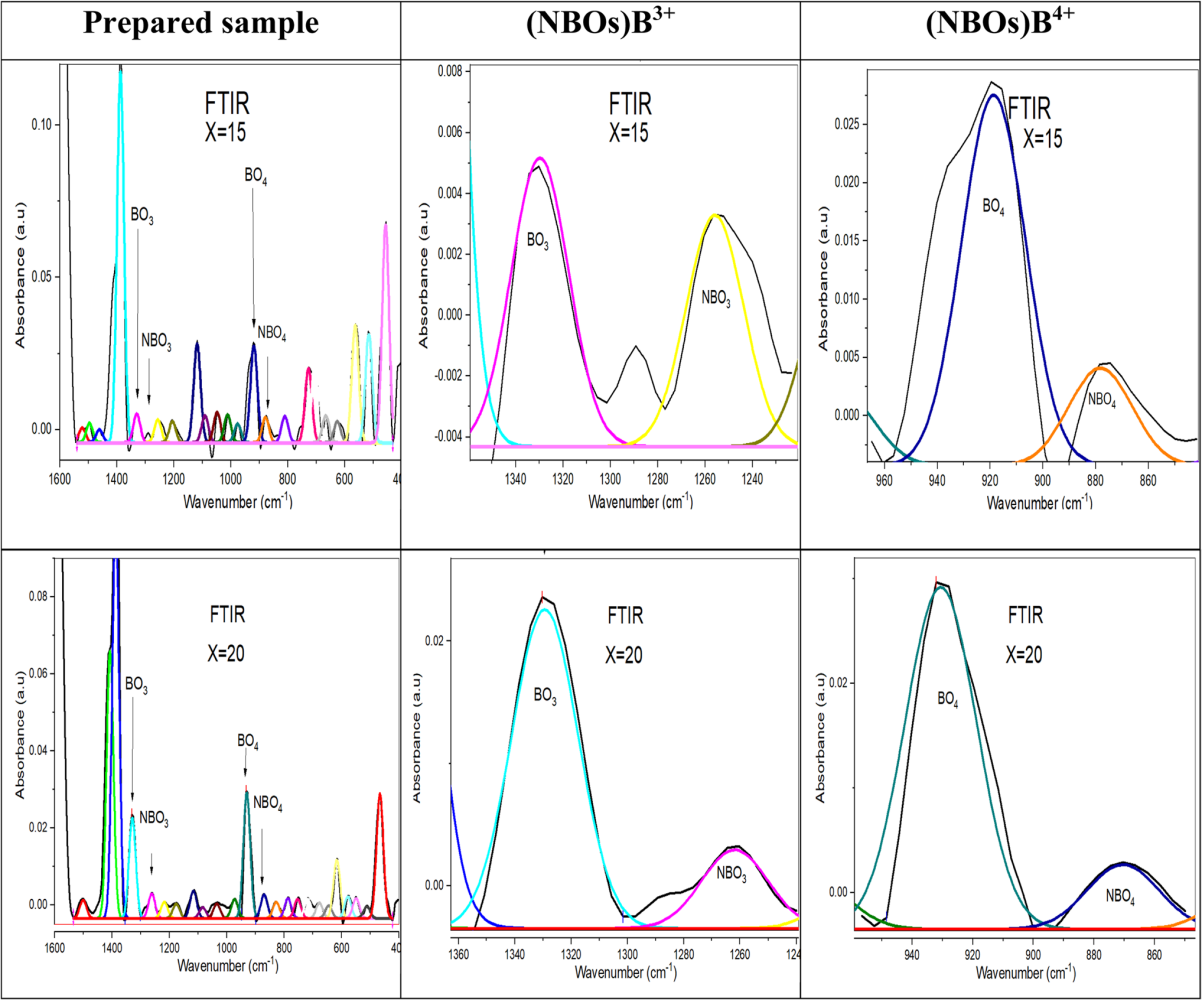
the deconvolution process of FTIR data of the prepared sample with the concentration of SrF_2_ for 50B_2_O_3_–(20–X) PbO–(X)SrF_2_–20CaO–10ZnO (where X = 0 and 5 mol%).

To analyze these bands as explicitly as possible, the deconvolution process was performed and well-displayed in Figs. 17, 18 and 19, based on FTIR analysis. Gaussian functions were used to precisely characterize the position of the absorption peaks and their intensity variation^27,29^.

The higher proportion of tetrahedral (B^4+^) units may be attributed to the ability of strontium fluoride to facilitate the formation of tetrahedral borate groups^29^. The smaller ionic radius of fluoride ions compared to oxygen ions allows for a more compact arrangement of atoms, enabling the boron atom to coordinate with four atoms, as confirmed by the values of the N4% ratio in Table 6^30^.

**Table 6:** The calculated N4% analysis for each glass sample under investigation.

Dopant concentration (mol)	N4 Ratio (%)	Trend
X = 0	33.82	Baseline (PbO-dominated)
X = 5	27.27	${F}^{-}$ disruption
X = 7.5	36.73	Partial recovery
X = 10	42.50	Threshold surge
X = 15	63.72	Sharp rise
X = 20	77.90	B^4+^ dominance

N4 is defined as the fraction of fourfold coordinated boron, N4 = B^4+^ / (B^3+^ + B^4+^), where B^4+^ is the number of tetrahedral borate units, also B^3+^ is the number of trigonal borate units^5,6,33,34^.

**Fig. 20:**
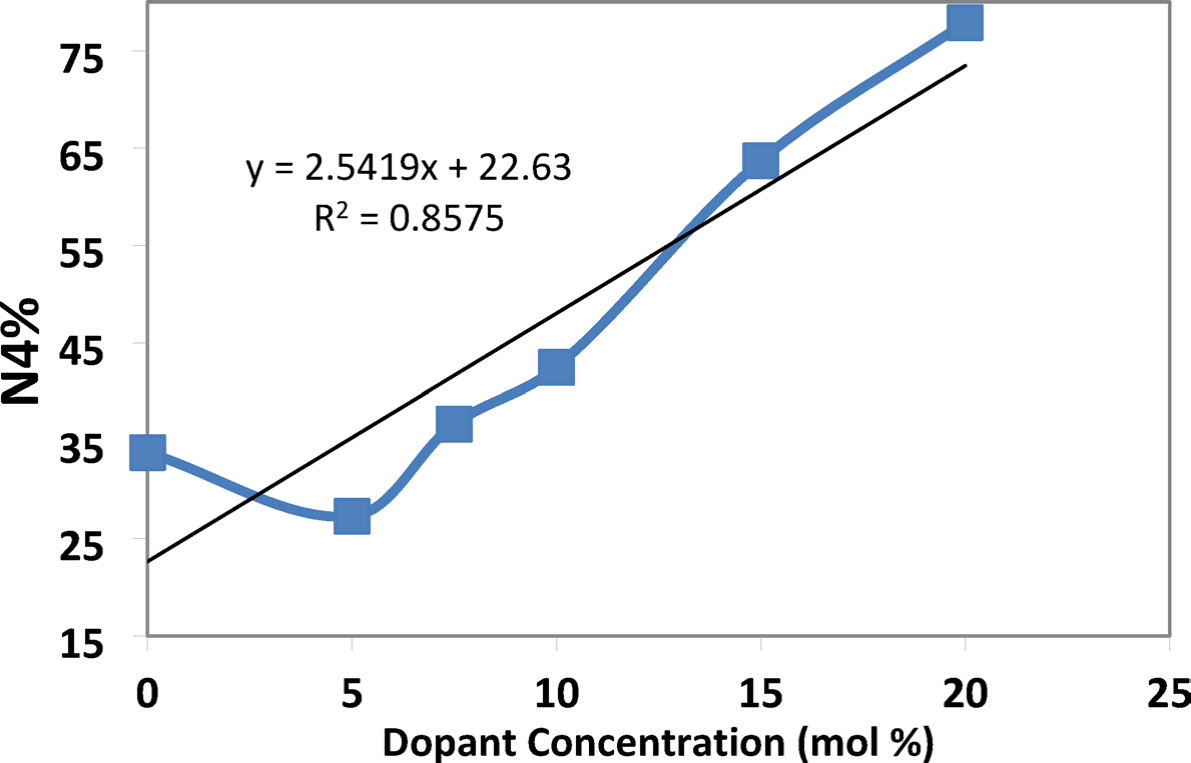
the N4% values with the concentration of SrF_2_ for 50B_2_O_3_–(20–X) PbO–(X)SrF_2_–20CaO–10ZnO (where X = 0 and 5 mol%).

The measured change in N4% value is obtained with the addition of strontium fluoride, discussed in Table 6. There is a decrease in the N4% curve, at X = 5, resulting from replacing some PbO with SrF_2_. Since ${\text{F}}^{-}$ It is monovalent; it can’t act as a bridging ligand like O^2−^ instead, it terminates B-O chains, leading to a reduction in the oxygen available for the formation of (B^4+^), as shown in Fig. 20^34^.

However, a critical transformation occurs at a strontium fluoride concentration of X = 7.5 as ${F}^{-}$ has a dual role where it not only compensates modifiers but also alters the network topology for being the most strong oxidizing agent. This transformation leads to a develop the development of B^4+^ tetrahedral units despite lower PbO^30^.

At higher concentrations of strontium fluoride, structural rearrangements occur due to fluoride saturation. So, fluoride ions start acting more as charge compensation rather than network disruptors, leading to B^4+^ stabilization. Additionally, Sr^2+^ has a stronger cation field strength compared to Pb^2+^, making it more effective at charge-compensating B^4+^ units.

**Fig. 21:**
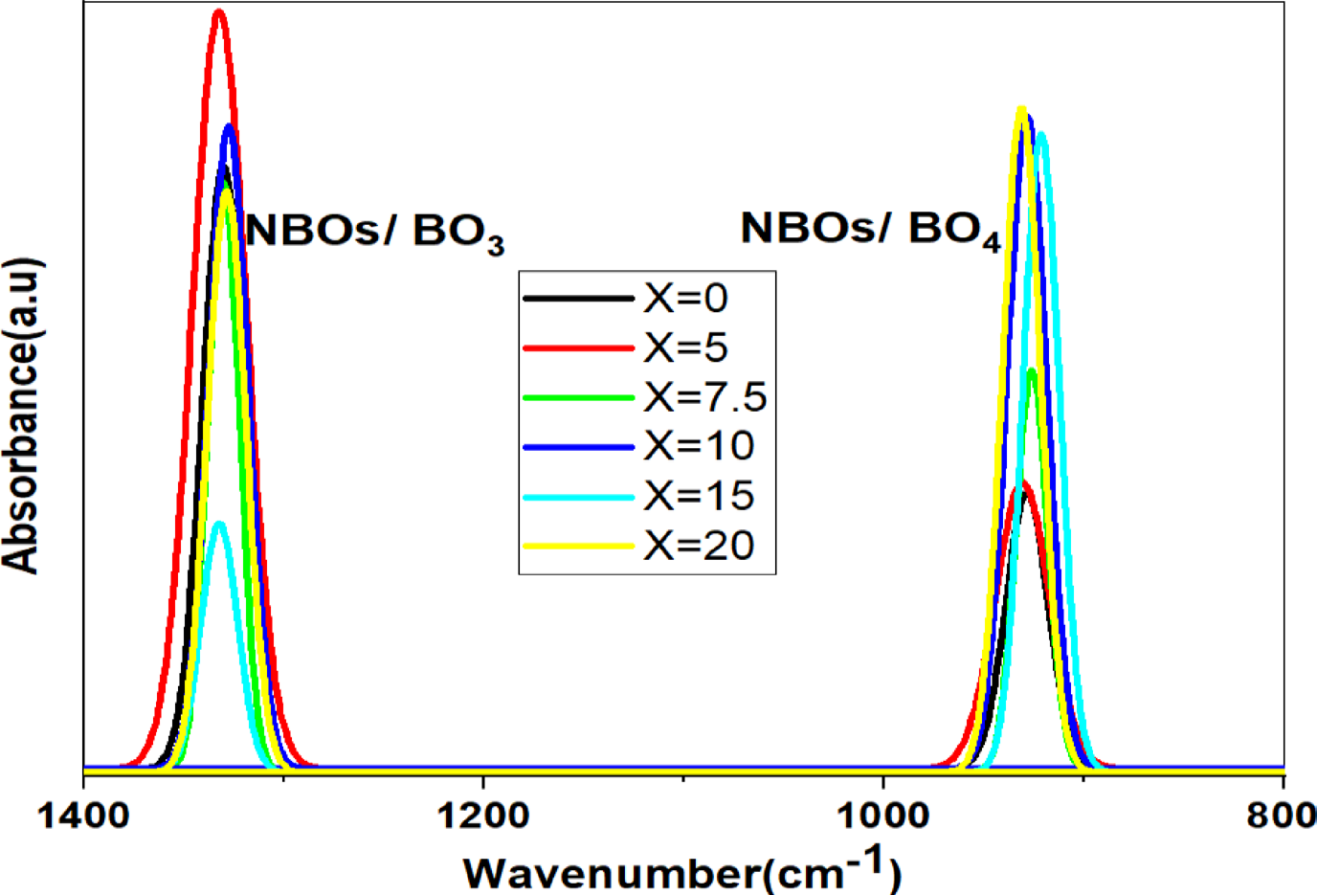
The effect of the addition of SrF_2_ on both (NBOs) B^4+^ and (NBOs) B^3+^ for 50B_2_O_3_–(20–X)PbO–(X)SrF_2_–20CaO–10ZnO (where X = 0, 5, 7.5, 10, 15, and 20 mol%).

As shown in Fig. 21, the increase in (NBOs) either with increasing tetrahedral B^4+^ units or decreasing trigonal B^3+^ units due to the presence of the strontium Sr^**2+**^ ion and fluoride ${\text{F}}^{-}$ ion for breaking the bond in B-O-B linkages and create B-F, B-O-Sr-F bonds as well as N-B-O^28,32^.

Notably, the number of non-bridging oxygen atoms decreased at high SrF_2_ percentages (15 and 20%). Where the network reaches a saturation state, it cannot accommodate additional fluoride ions. To enhance the formation of fluoroborate clusters (Fluoride-rich regions), segregate ${\text{F}}^{-}$ from the network. This frees ${\text{O}}^{2-}$ to form bridging bonds^32^. As well as strontium ions Sr^2+^ stabilize fluorinated units (${{\text{BO}}_{3}\text{F}}^{-}$,${{\text{BO}}_{2}{\text{F}}_{2}}^{-}$) by charge compensation, increase the tetrahedral borate units (B^4+^).

#### Ultrasonic Velocities measurements

The Young’s modulus (E) reveals threshold mechanics and indicates a complex relationship between atomic-scale structure evolution, compositional change, and mechanical performance; thus, the four parameters of mechanical measurements are obtained. The longitudinal, shear, bulk and Young’s elastic modulus of the glass samples are as follows^35,36^:

Longitudinal modulus: $$L={v}_{L}^{2}\times \rho$$

Shear modulus: $$G={v}_{S}^{2}\times \rho$$

Bulk modulus: $$K=L-(\frac{4}{3})\times G$$

Young modulus: $$E=2G\times (1+\sigma )$$

Poisson′s ratio: $$\sigma =\frac{L-2G}{2(L-G)}$$

According to the TCT model, an increase in the N4% ratio should lead to an increase in the constraint per atom. Consequently, an increase in the SrF_**2**_ concentration should make the system more rigid, resulting in a higher Young’s modulus value.

The substitution of PbO with SrF_**2**_ generates a non-linear trend in Young’s modulus (E), as shown in Table 7. The increase of SrF_**2**_ concentration leads to an increase in the elasticity from 61.98 GPa (X = 0) to 83.25 GPa (X = 20), reflecting the rigidity of the network despite the density dropping. The increase in tetrahedral borate units (B^4+^) forming a 3D cross-linked network, which are more rigid than trigonal borate units (B^3+^).

**Table 7:** Calculated parameters of mechanical data for each glass sample under investigation.

X = (mol%)	Shear modulus (GPa)	Longitudinal modulus (GPa)	Poisso′s ratio	Bulk modulus (GPa)	Young’s modulus (GPa)
0	24.07	80.74	0.29	48.65	61.98
5	28.66	101.16	0.30	62.94	74.65
7.5	29.58	101.22	0.29	61.79	76.52
10	26.69	104.42	0.33	68.83	70.91
15	31.65	108.90	0.30	66.70	81.98
20	32.07	111.38	0.30	68.62	83.25

**Fig. 22:**
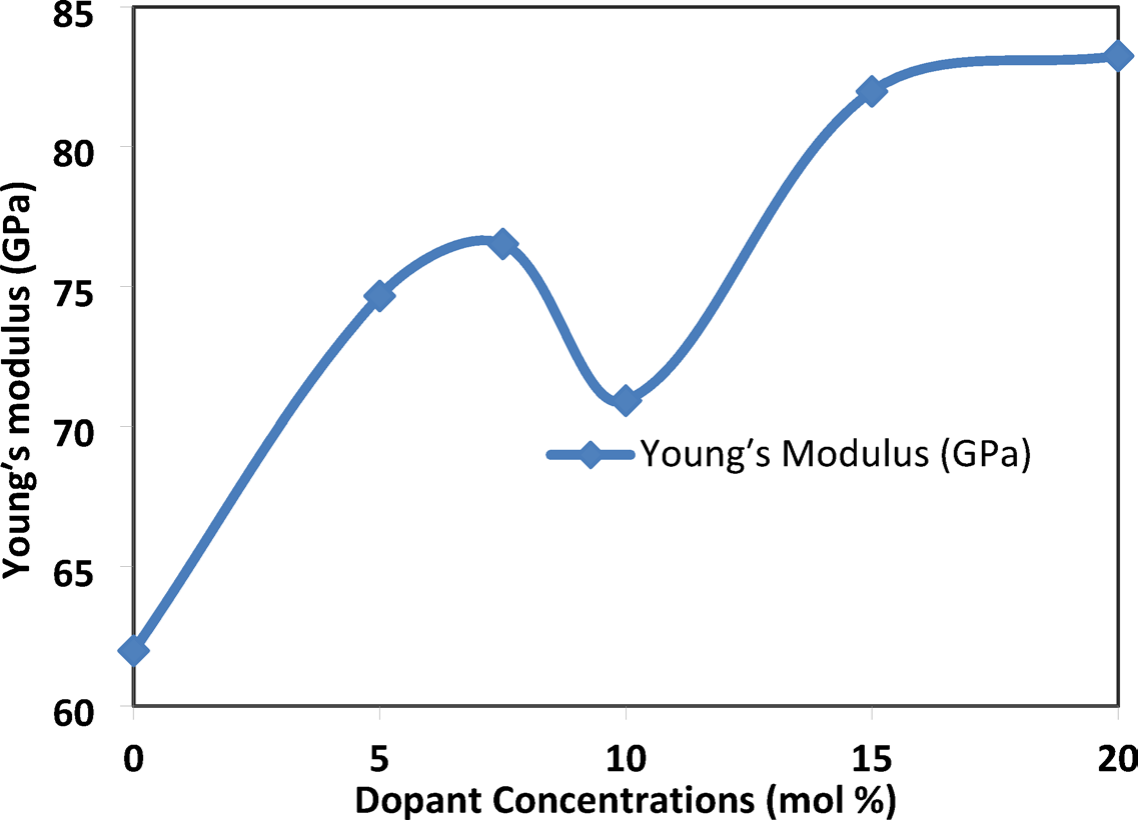
the effect of the addition of SrF_2_ on Young’s modulus for 50B_2_O_3_–(20–X) PbO–(X)SrF_2_–20CaO–10ZnO (where X = 0 and 5 mol%).

The anomalous borate units, fluorinated units (BO_3_F, BO_2_F_2_) defects and clusters formed at SrF_**2**_ concentration X = 10 mol% can cause a huge decrease in the value of elasticity despite the increase in the N4% ratio, as shown in Fig. 22.

### Percolation threshold: critical transitions

The percolation threshold represents a critical phase transition where F⁻ saturates and switches roles. The experimental data confirm three signatures of percolation-driven transformation₂ where:-

- Young’s modulus unexpectedly decreases to 70.91 GPa at SrF_2_ concentration X = 10 mol% (despite the increase in N4% (from X = 5 mol% to 10 mol%) as well as the longitudinal modulus increase), a result of the dual-role conflict at saturation (Fig. 22).
- N4% fraction threshold (X = 10 mol%): where F⁻ saturation triggers cooperative BO_4_ stabilization. FTIR evidence: BO_4_⁻/BO_3_ peak area ratio jumps from X = 10 to X = 15 mol%, Fig. (20).
- Density-Volume Decoupling where the density decrease monotonic (from 4.65 to 3.17 g/cm^3^) but molar volume shows Local maximum at X = 7.5 mol% (24.32 cm^3^/mol) and contraction at X = 10 mol% (23.76 cm^3^/mol), Fig. 2.

## Conclusion

This study resolves the century-old “boron anomaly” by a novel integration of MRN, TCT, and Percolation theories, revealing how SrF_2_ substitution in borate glasses simultaneously reduces density and enhances rigidity. Key insights include:

1. MRN demonstrates phase separation into ${\text{F}}^{-}$-rich disordered regions (45° XRD hump) and B^4+^-rich ordered domains (28° hump), explaining the coexistence of increased disorder (Urbach energy) and rigidity (direct bandgap).
2. TCT quantifies the B^3+^ to B^4+^ conversion (N4% increase), driving BO₄ tetrahedral percolation and rigidity (Young’s modulus increase despite the density reduction).
3. Percolation theory identifies a critical threshold at 10 mol% SrF_2_, where F⁻ saturation triggers a switch from network disruption (causing non-monotonic N4% and Young’s modulus anomalies) to charge compensation (stabilizing BO_4_ units at X=20 mol%).

This triple-theory synergy not only decodes the boron anomaly but also provides a predictive framework for designing eco-functional glasses. The SrF_2_-modified system shows promise for Pb-free applications in radiation shielding, optical fibers, and bioactive implants, highlighting the transformative potential of theory-driven glass innovation.

## Data Availability

All data generated or analyzed during this study are included in this article.
